# Mammalian adaptation of influenza A(H7N9) virus is limited by a narrow genetic bottleneck

**DOI:** 10.1038/ncomms7553

**Published:** 2015-04-08

**Authors:** Hassan Zaraket, Tatiana Baranovich, Bryan S. Kaplan, Robert Carter, Min-Suk Song, James C. Paulson, Jerold E. Rehg, Justin Bahl, Jeri C. Crumpton, Jon Seiler, Michael Edmonson, Gang Wu, Erik Karlsson, Thomas Fabrizio, Huachen Zhu, Yi Guan, Matloob Husain, Stacey Schultz-Cherry, Scott Krauss, Ryan McBride, Robert G. Webster, Elena A. Govorkova, Jinghui Zhang, Charles J. Russell, Richard J. Webby

**Affiliations:** 1 Department of Infectious Diseases, St Jude Children's Research Hospital, 262 Danny Thomas Place, Memphis, Tennessee 38105-3678, USA; 2 Department of Experimental Pathology, Immunology and Microbiology, Faculty of Medicine, American University of Beirut, PO Box 11-0236 Riad El Solh, Beirut 1107 2020, Lebanon; 3 Department of Computation Biology, St Jude Children's Research Hospital, 262 Danny Thomas Place, Memphis, Tennessee 38105-3678, USA; 4 Departments of Cell and Molecular Biology and Chemical Physiology, The Scripps Research Institute, 10550 North Torrey Pines Road, MEM-L71, La Jolla, California 92037, USA; 5 Department of Pathology, St Jude Children's Research Hospital, 262 Danny Thomas Place, Memphis, Tennessee 38105-3678, USA; 6 School of Public Health, The University of Texas Health Science Center at Houston, 1200 Pressler Street, Houston Texas 77030 USA; 7 Joint Influenza Research Center (Shantou University Medical College & Hong Kong University), Shantou University Medical College, Shantou, Guangdong 515031, PR China; 8 Department of Microbiology and Immunology, University of Otago, PO Box 56, Dunedin 9054, New Zealand; 9 Department of Microbiology, Immunology & Biochemistry, College of Medicine, The University of Tennessee Health Science Center, Memphis, Tennessee 38163, USA

## Abstract

Human infection with avian influenza A(H7N9) virus is associated mainly with the exposure to infected poultry. The factors that allow interspecies transmission but limit human-to-human transmission are unknown. Here we show that A/Anhui/1/2013(H7N9) influenza virus infection of chickens (natural hosts) is asymptomatic and that it generates a high genetic diversity. In contrast, diversity is tightly restricted in infected ferrets, limiting further adaptation to a fully transmissible form. Airborne transmission in ferrets is accompanied by the mutations in PB1, NP and NA genes that reduce viral polymerase and neuraminidase activity. Therefore, while A(H7N9) virus can infect mammals, further adaptation appears to incur a fitness cost. Our results reveal that a tight genetic bottleneck during avian-to-mammalian transmission is a limiting factor in A(H7N9) influenza virus adaptation to mammals. This previously unrecognized biological mechanism limiting species jumps provides a measure of adaptive potential and may serve as a risk assessment tool for pandemic preparedness.

## Abstract (web summary)

H7N9 bird flu viruses cause mild disease in poultry but can occasionally infect humans with fatal consequences. Here, the authors show that viral genetic diversification is low in ferrets and high in chickens, suggesting that a genetic bottleneck limits H7N9 adaptation to mammals

## Graphical abstract

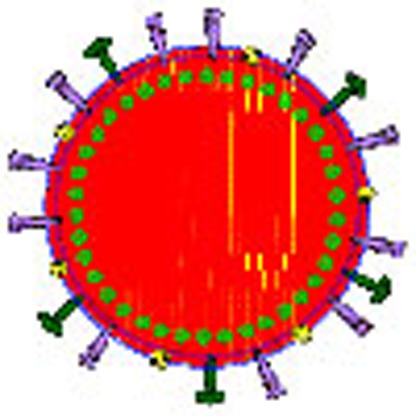

In February 2013, avian influenza A(H7N9) virus crossed the species barrier in China and for the first time caused human infections^1^. As of November 2014, 457 human infections had been reported, with a 30% case fatality rate^2^. Epidemiological evidence has linked human cases with exposure to live-bird markets^3^,^4^,^5^. Closure of these markets, with the likely assistance of environmental factors, helped to halt the first wave (February–May, 2013) of the outbreak^6^. However, public health efforts failed to completely stop the spread of human H7N9 infections; more cases were reported as the second wave began in October 2013 (ref. 7) and has continued to date^2^.

Sporadic clusters of H7N9 virus transmission among family members have been reported^8^,^9^,^10^; however, as yet there is no evidence of sustained human-to-human transmission. Previous studies have shown that airborne H7N9 virus transmission can occur, albeit with low efficiency in ferrets^11^,^12^,^13^,^14^, the standard model used to simulate transmissibility among humans^15^. Several viral factors can contribute to the adaptability of avian-like viruses to mammals^16^: virus morphology^17^, receptor-binding specificity^18^, changes in glycosylation patterns of the hemagglutinin (HA)^19^, polymerase activity^20^, the acid stability of the HA^21^ and the HA-neuraminidase (NA) balance^22^. Variation in these traits may be controlled by only a few amino-acid substitutions in a virus genome^17^,^20^,^21^,^22^. Only a few amino-acid changes are required to allow an avian-adapted virus to become airborne-transmissible between mammals, as was demonstrated in the influenza A(H5N1) viruses^23^,^24^.

To elucidate the transmission dynamics and transmission barriers of the emerging H7N9 influenza viruses, we evaluated the replication efficiency, genomic diversification and host adaptation of A/Anhui/1/2013 (H7N9) influenza virus after the inoculation of chickens and ferrets and during ferret-to-ferret transmission. Here we provide evidence that H7N9 virus infection of chickens, while asymptomatic, promotes genetic diversity in the virus population, increasing the likelihood of substitutions that favour mammalian infection and transmission. Conversely, the virus shows poor airborne transmissibility and no evidence of further adaptation upon replication in ferrets, likely due to a narrow bottleneck constraining genetic diversification.

## Results

### Replication and genetic diversity in chickens

The H7N9 virus was isolated mainly from chickens in live-bird markets suspected to be the source of human infections. Thus, we investigated the virulence and replication of A/Anhui/1/2013 (H7N9), one of the first human H7N9 viruses isolated, in chickens. A/Anhui/1/2013 shares at least 99% identity at the nucleotide and protein levels with H7N9 influenza viruses isolated from birds and live-bird markets (Supplementary Table 1).

**Figure 1:**
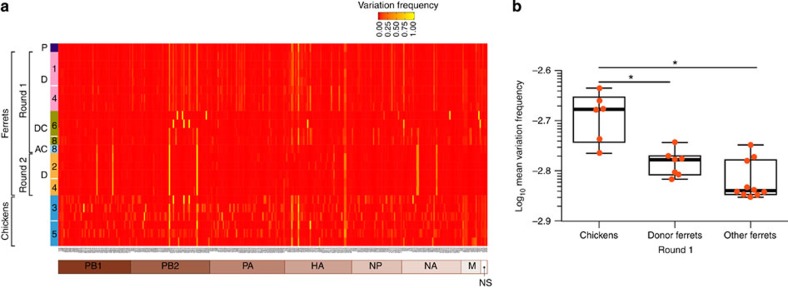
Mutation frequency in A/Anhui/1/13 (H7N9) influenza viruses collected from chickens and ferrets. (**a**) Heat map showing nucleotide substitutions identified in chicken (oropharyngeal swabs) and ferret (nasal washes) virus isolates by using Illumina Mi Seq. Only mutations with a frequency >0.03 are shown. Numbers on the *y* axis represent the day post inoculation. (**b**) Mean variation frequency. Mean variation frequency for each sample was calculated using positions that are variable in at least one of the examined samples. If a site has a minor variant frequency above zero in any of the samples this was included in the nominator and the denominator of the proportion was the total number of variable sites that was variable in all samples. Variation frequency was expressed as the negative log. ‘Donor ferrets Round 1' are ferrets inoculated with parental A/Anhui/1/13 (H7N9) influenza virus; ‘Other ferrets' are contact ferrets in round 1 of experiments and donor ferrets in round 2. *P<0.05, two-tailed Mann–Whitney *U-*test. P, parental virus; D, donor; DC, direct contact; AC, airborne contact.

After intravenous injection with 10-fold dilutions of A/Anhui/1/2013 virus stock, none of the groups of 10 chickens showed signs of illness or succumbed to infection, indicating an intravenous pathogenicity index of 0. We then determined the extent and duration of virus shedding in groups of four chickens inoculated with the A/Anhui/1/2013 virus via the natural routes (nares, trachea and eyes). Inoculated chickens showed no signs of illness but shed on average 3.75 log_10_EID_50_ (50% egg infectious doses) per ml of virus from the upper respiratory tract at 3 days post inoculation (dpi), and shedding lasted for as long as 7 dpi (Table 1), suggesting that they can sustain H7N9 virus in the environment with little burden to their health. We then investigated the extent of genetic diversity of H7N9 viruses after replication in chickens. Deep sequencing of viral RNA from oropharyngeal swabs of inoculated birds revealed many synonymous and non-synonymous mutations (see ‘chickens', Fig. 1a; Supplementary Data 1). All the gene segments except NS accumulated multiple amino-acid substitutions (Supplementary Table 2), although the HA genetic pool observed in the egg-grown parental stock was more or less maintained. The substantial genetic diversity, together with protracted asymptomatic virus shedding and close human-poultry contact in live-poultry markets, provide an optimal environment for the generation of viruses with the ability to infect humans.

### Virulence, transmission and adaptation in ferrets

**Figure 2:**
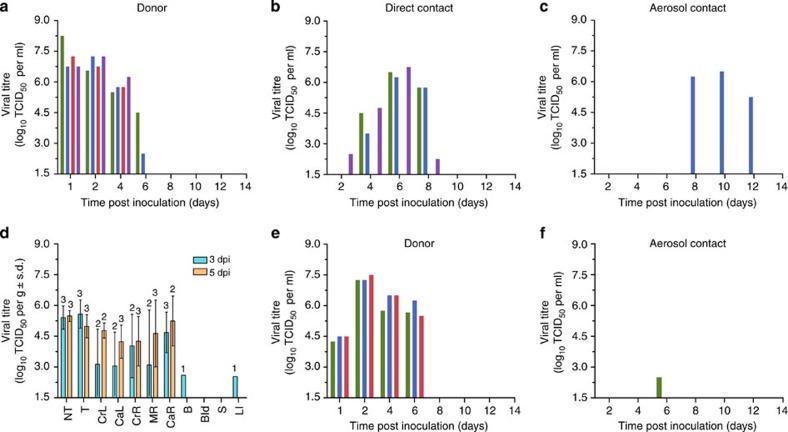
Replication and transmission of A/Anhui/1/2013 (H7N9) influenza virus in ferrets. (**a**–**c**) Nasal wash virus titres in animals during round 1 experiment. Coloured bars represent individual ferrets within each group. (**d**) Viral titres in the tissues collected from H7N9-infected ferrets on the indicated days post inoculation. The numbers of tissue samples with virus titres higher than the limit of detection (1.5 log_10_TCID_50_ per gram, *n*=3) are indicated above each bar (**e**,**f**). Nasal wash titres in round 2 experiment in which the donors were inoculated with virus previously transmitted by airborne contact. Coloured bars represent individual ferrets within each group. NT, nasal turbinates; T, trachea; lung lobes: CrL, cranial left; CaL, caudal left; CrR, cranial right; MR, middle right; CaR, caudal right; B, brain; Bld, blood; S, spleen; LI, large intestine.

**Figure 3:**
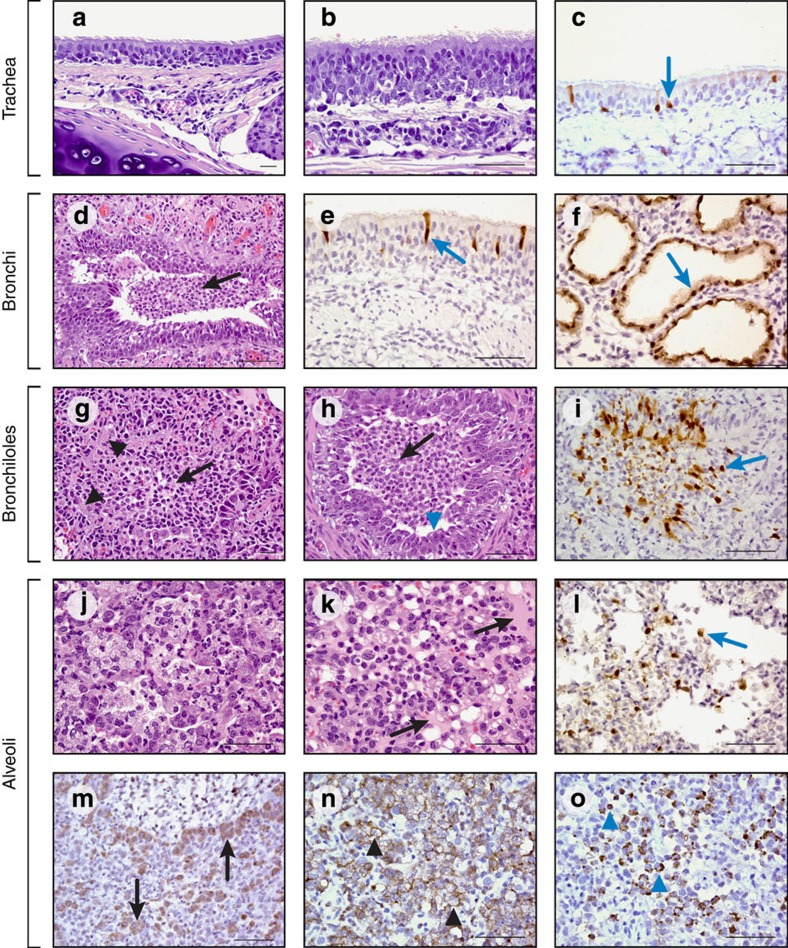
Histologic findings in the respiratory tracts of ferrets inoculated with A/Anhui/1/2013 (H7N9) influenza virus. Representative features observed in tracheas (**a**–**c**), bronchi (**d**–**f**), bronchioles (**g**–**i**) and alveoli (**j**–**o**) on day 3 (**a**,**d**,**g**,**h**,**i**,**j**,**k**,**n**,**o**) and day 5 (**b**,**c**,**e**,**f**,**l**,**m**) post inoculation. Stains were hematoxylin and eosin (**a**,**b**,**d**,**g**,**h**,**j**,**k**) or immunohistochemical stains for influenza A virus nucleoprotein (**c**,**e**,**f**,**i**,**l**), pneumocyte type II cells (**m**; surfactant protein **c**), macrophages (**n**; ionized calcium-binding adapter molecule **1**), and neutrophils (**o**; myeloperoxidase). Influenza A was detected in tracheas (**c**), bronchi (**e**), submucosal glands (**f**), bronchioles (**i**) and alveolar epithelial cells (**l**) (blue arrows). (**a**–**c**) Tracheas showed multifocal epithelial hyperplasia (**b**) and mucosal and submucosal (**a**,**b**) neutrophil and lymphocyte infiltration. (**d**–**f**) Bronchi showed multifocal epithelial hyperplasia, submucosal gland epithelial necrosis, macrophage and neutrophil infiltration, and luminal macrophages, neutrophils and cellular debris (d, black arrow). (**g**–**i**) Bronchioles showed epithelial necrosis (**g**, black arrowheads), regenerative hyperplasia (**h**, blue arrowhead), and marked luminal macrophage, neutrophil and lymphocyte infiltrates admixed with cellular debris (**g**,**h**, black arrows). (**j**–**o**) Peribronchiolar alveoli (**j**,**k**) had varying degrees of pneumocyte necrosis and regeneration (type II hyperplasia; **m**, black arrows), macrophage infiltration (**n**, black arrowheads), neutrophil infiltration (**o**, blue arrowheads), oedema (**k**, black arrow) and cellular debris. Scale bars, 20μ—**h**, **m**; 50μ—**a**,**b**,**c**,**e**,**f**,**g**,**i**,**j**,**k**,**l**,**n**,**o**; 100μ—**d.**

We next investigated the virus's virulence and its transmissibility among ferrets via contact and airborne routes. Inoculated ferrets displayed only transient weight loss and fever (Supplementary Fig. 1) despite substantial (average 6.95 log_10_ TCID_50_ (50% tissue culture infectious doses) per ml of virus on 2 dpi) and protracted (maximum, 6 days) virus shedding from the upper respiratory tract, as determined in nasal washes (Fig. 2a). Lung virus titres peaked 5 dpi at an average of 10^5^ TCID_50_ per gram, indicative of substantial lower respiratory tract infection (Fig. 2d). Histological analysis revealed mild tracheitis, bronchitis with submucosal gland involvement and bronchoalveolar pneumonia (Fig. 3). Minor virus replication was detected in the brain and large intestine in one of the three inoculated ferrets (Fig. 2d). Virus was transmitted from inoculated (donor) ferrets to three of the four cage-mates by direct contact (DC) within 1–2 days and to one of the four ferrets by airborne contact (AC) within 7 days (Fig. 2b,c). Virus titres and duration of shedding were comparable in ferrets infected by DC and AC. All the four DC ferrets and two of the four AC ferrets became seropositive (Supplementary Table 3). This limited mammalian transmission by respiratory droplets is consistent with epidemiologic reports of limited human-to-human transmission of H7N9 virus in only a few familial clusters^10^ and with data from other ferret studies^11^,^12^,^14^,^25^,^26^.

A second round of infection was then conducted to investigate the properties of the airborne-transmitted H7N9 virus in ferrets. We inoculated three donor ferrets with ‘AC' virus obtained from the nasal wash of the ferret initially infected by airborne transmission. To further characterize the AC virus, we measured the level of its replication in donor ferrets and its airborne transmissibility. Ferrets inoculated with AC virus shed virus at titres similar to those of the ferrets inoculated with the parental (stock) virus except at 1 dpi, when the titre was much lower in ferrets inoculated with AC virus despite equivalent inoculum (Fig. 2e). Virus was shed only transiently and minimally by one of the three secondary airborne-contact ferrets, at 5 days post contact (Fig. 2f). These findings demonstrated inefficient secondary airborne transmissibility and poor further adaptation towards this trait of the H7N9 virus during the previous round of replication in a ferret host.

### Genetic diversity of H7N9 viruses in ferrets

To compare the genetic diversity generated during replication of H7N9 virus in a mammalian versus an avian host and to screen for selection of amino-acid substitutions after airborne transmission in mammals, we performed deep sequence analysis of viral RNA isolated from ferret nasal washes during both rounds of the experiments. Overall, viral RNAs from ferrets were significantly less diverse (*P*<0.05, two-tailed Mann–Whitney *U-*test) than those isolated from chickens (Fig. 1b, Supplementary Table 4, Supplementary Data 2) suggesting the existence of a constraint, or narrow bottleneck, in genetic diversification during replication in mammals. On comparing the genetic diversity of the surface glycoproteins, we found significantly less genetic diversity in the HA gene in ferrets than in chickens, but no significant difference between the diversity of the NA genes (Supplementary Fig 2). These results suggest that a strong purifying selection pressure was imposed on the HA gene during replication in ferrets. At the amino-acid level, four variant residues (PB1-D76N, NP-I365V, NA-E73K and -I300V; N9 numbering) occurred at a high frequency (≥40%) in the AC isolate and were maintained in ferrets inoculated with AC virus (Supplementary Fig 3b,e and f), Supplementary Table 5).

### Effect of selected substitutions on protein function

**Figure 4:**
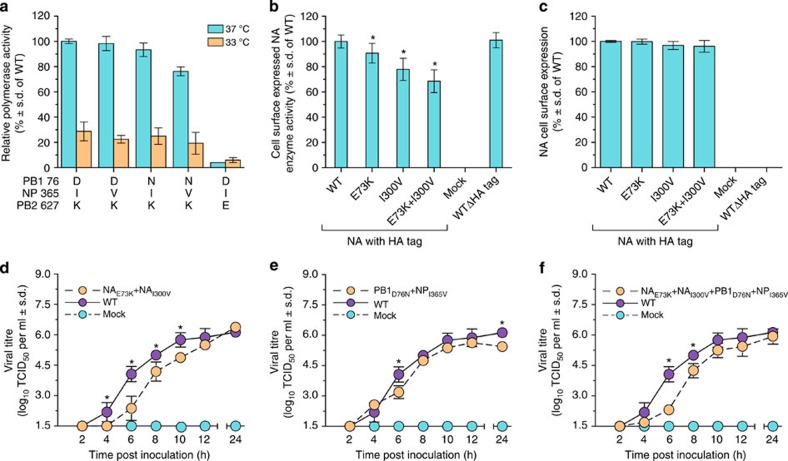
Effect of identified amino-acid substitutions in the NA, PB1 and NP on protein function and replication kinetics of recombinant A/Anhui/1/2013 (H7N9) influenza viruses *in vitro.* (**a**) Effect of temperature on polymerase complex activity with the indicated amino-acid substitutions. (**b**) Effect of the indicated amino-acid substitutions on NA activity. (**c**) Cell surface expression of the NAs shown in panel **b**. (**d**–**f**) Replication kinetics of rg-A/Anhui/1/2013 (H7N9) influenza viruses with the indicated amino-acid substitutions in the NA, PB1 and NP genes in MDCK cells. Values are mean and s.d. from two independent experiments (*n*=4). *P<0.05 compared with WT virus by two-way analysis of variance.

We next examined the effect of substitutions present at high frequency after airborne transmission on virus polymerase and NA activity *in vitro.* In minigenome replication assays, the dual PB1-D76N and NP-I365V substitutions reduced the polymerase activity by 23%, but single substitutions had no effect (Fig. 4a). Overall, the polymerase activity of both the parental and mutant polymerase complexes was significantly lower at 33 °C (human upper respiratory tract temperature) than at 37 °C (avian upper respiratory tract temperature) despite the presence of the mammalian-adaptive PB2-E627K substitution^27^. Reversing the substitution to the avian-like PB2-627E reduced polymerase activity markedly (to 6–8% of wild-type (WT) activity). The NA-I300V substitution reduced NA activity by 23% (by 33% when combined with NA-E73K) without altering its cell surface expression (Fig. 4b,c). The negative effects of these mutations on virus protein functions suggest that these residue changes either were present in the viral inoculum at low levels and their fixation during the transmission study was somewhat stochastic or that they had ‘hitchhiked' along with adaptive mutations.

### Replication kinetics of recombinant viruses *in vitro*

The observed reduction in polymerase activity in the minireplicon assay and the decreased NA activity *in vitro* suggests that an influenza virus carrying the identified amino-acid substitutions would be attenuated in cell culture. To assess this possibility we generated recombinant (rg) A/Anhui/1/2013 influenza viruses with single or multiple amino-acid substitutions in the gene/s of interest and compared their replication kinetics with the WT rg-virus in Madin Darby canine kidney (MDCK) cells. In line with the drop in NA and polymerase enzymatic activities, rg-A/Anhui/1/2013 (NA_E73K_+NA_I300V_), rg-A/Anhui/1/2013 (PB1_D76N_+NP_I365V_) and rg-A/Anhui/1/2013 (NA_E73K_+NA_I300V_ +PB1_D76N_+NP_I365V_) influenza viruses replicated to significantly lower titres than the respective WT strain (Fig. 4d,e and f, respectively); and the total amount of virus produced over the course of experiment, as quantified by the area under virus titre hour curve from 0 to 24 h (AUC_0–24_), was significantly lower when compared with the WT virus (AUC_0–24_ 71.13; 72.25; 69.19 versus 80.38 log_10_TCID_50_ per ml, respectively, *P*<0.05; Table 2). These data confirmed our loss of function expectations. Viruses with the individual amino-acid substitution in the NA, rg-A/Anhui/1/2013 (NA_E73K_) and rg-A/Anhui/1/2013 (NA_I300V_) viruses were significantly attenuated *in vitro* (Supplementary Fig. 4a,b), as demonstrated by the total amount of virus produced over the course of the experiment (AUC_0–24_ 63.88; 76.20 versus 80.38 log_10_TCID_50_ per ml, respectively, *P*<0.05; Table 2). Virus titres and AUC_0–24_ values for rg-A/Anhui/1/2013 (PB1_D76N_) and rg-A/Anhui/1/2013 (NP_I365V_) were comparable to those of the WT virus at 37 °C (Supplementary Fig. 4c,d; Table 2). Therefore, the observed attenuation in growth kinetics in MDCK cells could be largely attributed to the NA_E73k_ mutation.

Further, given these results and the minimal infection that followed secondary airborne transmission, we concluded that the combination of these substitutions attenuates virus replication *in vitro* and does not contribute to viral transmission fitness and that the H7N9 virus does not further adapt to mammalian hosts upon limited replication and transmission chains.

### Receptor-binding specificity

We detected six variant HA residues (Supplementary Table 5) in the stock parental A/Anhui/1/2013 (H7N9) virus, including the L235Q mutation (L217Q in H3 numbering), which confers a human-to-avian shift in receptor-binding preference^25^. The frequency of the L235Q variant increased to ∼90% in one chicken isolate but was undetectable in four of the six chicken specimens analysed. Conversely, and against expectations, the L235Q mutation increased (albeit slightly) in frequency in ferrets inoculated with the parental virus but decreased in frequency among the DC ferrets. This mutation became undetectable in the AC ferret and in ferrets inoculated with AC samples, suggesting that the L235Q variant is not efficiently transmitted in ferrets. In addition, three of the six variants were completely undetectable in ferrets inoculated with AC virus.

**Figure 5:**
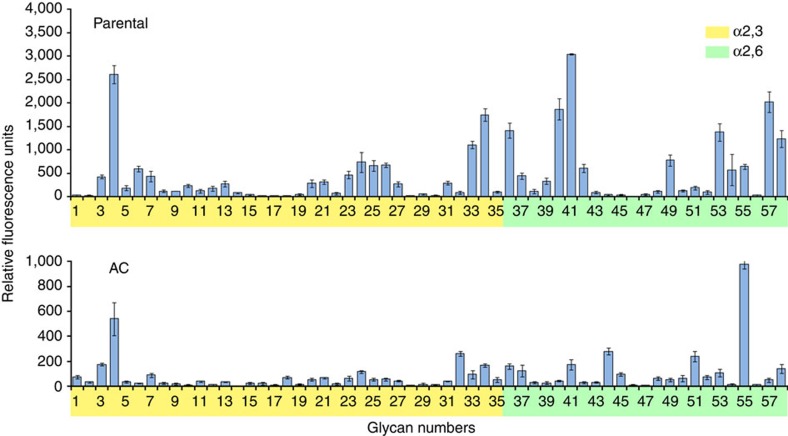
Receptor-binding preference of A/Anhui/1/2013 (H7N9) influenza virus. Glycan microarray containing α2,3 and α2,6 sialosides was used to compare the receptor-binding affinity of parental and AC (isolated on day 8 post inoculation after aerosol transmission) A/Anhui/1/2013 (H7N9) influenza virus. Values are the means±s.d. of six replicate spots per glycan.

We then compared the binding preference of the parental and airborne-transmitted (AC) viruses to a wide array of glycans (Fig. 5). Both the viruses bound both avian (α-2,3–) and human (α-2,6–) sialic acid receptors, but the AC virus' affinity to the both receptors was markedly reduced. This drop in binding affinity may be due to the loss of several HA variants originally present in the parental stock or due to the reduction in NA activity; the cause remains to be investigated.

### Acid stability of wild-type H7N9 influenza viruses

Acid stability has been described as one of the markers for regulating host range and virulence of influenza viruses^21^,^23^,^28^. We analysed the optimal pH at which the HA protein induces syncytia formation in infected Vero cells. The pH of HA activation of the three H7N9 viruses tested ranged between 5.6 and 5.8 (Supplementary Fig. 5). Thus, the HA proteins from these viruses are relatively unstable compared with those from human influenza viruses that are typically activated at pH 5.0–5.2 (refs 29, 30). The HA activation pH values for the H7N9 viruses are more similar to those reported for the highly pathogenic avian H5N1 influenza viruses^31^,^32^.

## Discussion

Our study revealed a previously unknown population-level factor restricting the further adaptation of avian A(H7N9) influenza virus to mammals. Although the H7N9 virus replicates to high titres in infected ferrets, replication in this host did not select for viruses with enhanced growth, virulence or transmissibility. In contrast, our data suggest that replication in ferrets lead to an attenuated phenotype. Because adaptation depends on the extent of the influenza virus population's diversity in the host, we propose the existence of a ‘genetic bottleneck' that limits the ability of avian A(H7N9) influenza virus to adapt to a mammalian host by limiting its genetic diversity in that host. By using a population-level approach and comparing the genetic diversity of the virus population in the natural avian host and in infected mammals, we found that the genetic diversity of A/Anhui/1/2013 (H7N9) influenza virus was limited after its transmission to mammals. The loss in genetic variation imposed by the narrow genetic bottleneck appears to restrict the capacity of A(H7N9) virus to fully adapt to mammals.

Genetic bottlenecks occur frequently during the natural life cycles of RNA viruses^33^. Every RNA virus, including influenza A virus, exists as a heterogeneous genetic pool. This genomic diversity is an important component of influenza virus adaptation^34^. The genomic diversity of the inoculum population, the diversity generated during infection and the diversity transferred during airborne transmission contribute to adaptation to a given host^16^,^35^. A fully mammalian-adapted influenza virus can cause upper respiratory infection in humans and can be transmitted via the airborne route; the H7N9 virus appears to have the capacity for the former but not the latter. Avian-origin influenza viruses have the potential to cross the species barrier and cause influenza pandemics in humans, depending on their adaptive capacity. Our results are consistent with the hypothesis that the adaptive potential of the A(H7N9) virus population is limited by the loss in genetic variation from the tight genetic bottleneck imposed by selection in the mammalian host environment. For any given avian influenza virus, a combination of genetic traits must be selected to enable the adaptation to mammalian hosts. While a subpopulation of virions in a host may possess some of these factors^23^,^24^, the genetic bottleneck imposed on the virus population remains a barrier to the selection and survival of those variants.

Several pieces of evidence, including the isolation of the H7N9 virus from chickens at live-bird markets and the exposure history of infected patients, suggest that live-poultry markets are a source of human infection^4^,^5^,^36^,^37^. Specifically, chicken layers and quail have been shown to be susceptible to H7N9 influenza virus infection and to shed virus at much higher titres than other avian species, including pigeons and Pekin ducks^38^. Closure of live-bird markets in China temporarily halted the H7N9 outbreak^39^. After the markets reopened and the winter season began, a new wave of infection emerged. Nearly 460 human cases had been reported as of November 2014 (ref. 2), raising new concern about the pandemic threat posed by this virus.

Our data show that the H7N9 virus replicates in chicken with moderate efficiency and that the infected chickens shed virus for a protracted period. This shedding appears to be sufficient to allow contact transmission of H7N9 virus among chickens, although with poor efficiency^38^. The ability of the H7N9 virus to replicate in chickens is likely aided by its internal gene cassette, derived from the H9N2 viruses of chicken origin^40^. The silent (asymptomatic) replication of the virus in chickens results in undetected spread of the H7N9 virus in the poultry markets and increases the risk of human infection through exposure to infected, asymptomatic chickens. Other bird species may play a role in spread of the H7N9 virus between bird markets and across provinces. One study showed that wild birds, including finches, sparrows and parakeets, support replication of H7N9 viruses^41^. The diversity generated through replication in these hosts and the potential role they play in H7N9 outbreaks remains to be investigated.

The human H7N9 isolates possess, or can readily acquire, several traits that support mammalian adaptation, including the PB2-E627K mutation^42^, which enhances replication in the mammalian upper respiratory tract, promoting virulence and airborne transmissibility^24^,^27^,^43^,^44^, and the HA-Q235L substitution (226 in H3 numbering)^45^, which confers human-like receptor preference. Indeed, the H7N9 virus replicated to titres similar to those of human H1N1 virus (A/Brisbane/59/2007; ref. 46) in the upper respiratory tracts of ferrets and was transmitted to contact ferrets at a similar rate, albeit with lower efficiency. In contrast, other avian-origin influenza viruses, such as H5N1 virus, are not transmitted among ferrets even by DC^21^,^47^,^48^. However, our results show that the mammalian transmissibility of H7N9 virus may deteriorate rapidly and is not sustained. Overall, the poor efficiency of H7N9 airborne transmissibility is consistent with previous reports^11^,^25^,^26^. The low transmission efficiency can be attributed to several key properties of the H7N9 virus: (1) mixed preference for both avian and human receptors; (2) suboptimal acid stability, with a fusion pH optimum higher than that reported for human-transmissible viruses^23^,^29^,^49^; and (3) reduced polymerase activity at the human upper respiratory tract temperature (33 °C).

The presence of a significant subpopulation of variants with the HA mutation Q235L, which confers human receptor-binding preference^25^, among the human isolates did not appear to result in a switch to fully human receptor preference; the H7N9 virus maintained the binding affinity for both human and avian receptors. This result could be attributed to the presence of a subpopulation (23%) of viruses containing the avian Q235 variant or to the enhanced NA binding^12^. In the aerosol contact-transmitted (AC) virus population, the Q235 variant population fell to <10%; the virus maintained preference for both avian and human receptors, albeit with markedly reduced affinity, suggesting that other residues might be masking the effect of the Q235L mutation. Several variants (N141D, A143T and N167D; residues 132, 134 and 158, respectively, in H3 numbering) that were present in the parental virus fell to undetectable levels in the AC virus population and in ferrets inoculated with the AC virus. These residues are located in the 130- and 150-loops of the receptor-binding domain (ref. 45) and could potentially play a role in the receptor preference of H7N9 virus. Further studies are required to elucidate the effect of these residues on receptor-binding preference and avidity.

In this study, we found that H7N9 viruses that had been transmitted via the airborne route were not better adapted or more transmissible in mammals than the parental strain. Four residues in the three genes were uniquely enriched after airborne transmission, and in the ferrets inoculated with AC virus in round 2 experiments. Two of these substitutions (E73K and I300V) located in the NA gene resulted in a decrease in NA activity, and the PB1-D76N and NP-I365V mutations reduced polymerase activity. These mutations also attenuated virus replication *in vitro.* Importantly, although a human H7N9 virus was passaged in eggs to prepare a sufficient stock for experiments, the effect of egg-selected variants was controlled by using the same parental strain to inoculate chickens and ferrets. Richard *et al.*^11^ previously demonstrated the failure of H7N9 virus to adapt to ferrets due to the selection of variants with higher avian receptor-binding affinity, higher pH of activation and lower thermal stability after airborne transmission. None of the enriched variants in our study coincided with the ones described by Richard *et al.*^11^ or with sequence heterogeneity detected within specimens from human patients^50^. Thus, genetic selection of H7N9 virus after airborne transmission is a stochastic event and it does not favour mammalian adaptation or mammalian transmissibility.

In comparing the genetic diversity of the surface glycoproteins, we observed differences in genetic diversity among species in the HA gene but not in the NA gene. At the amino-acid level, none of the HA variants in the parental virus stock were detectable in the transmitted virus population. This result strongly suggests that a purifying pressure acting mainly on the HA gene (virus entry) shapes the diversity bottleneck observed in ferrets. This purifying pressure did not appear to select variants with novel traits such as enhanced transmissibility of the virus in ferrets. Similar purifying pressure on the HA gene has previously been described in association with the airborne transmission of H5N1 virus. However, in the case of H5N1 the purifying pressure appeared to favour the selection of viruses with enhanced fitness after aerosol transmission^51^, which was likely aided by the laboratory forced pre-selection of a receptor-matched virus mutant before the inoculation of ferrets^24^. In our study the diversity bottleneck acting on H7N9 virus appeared to persist even after a subsequent passage of the aerosol-transmitted virus in ferrets. Together, these findings suggest that the H7N9 influenza virus is not readily adaptable to transmit in mammals, which could partially explain the absence of sustained human-to-human transmission to date.

Despite their ability to infect humans, the currently circulating strains of H7N9 viruses did not readily acquire additional adaptive mutations during their limited passage in humans or a mammalian host like ferrets. Our results suggest that viral replication in chickens may enhance the diversity of the H7N9 viruses and increase the potential of a subpopulation of variants to enter the mammalian cells and replicate. Even though these variants can replicate in mammals, the stringency of the genetic bottleneck limits their ability to acquire subsequent mutations that would allow mammal-to-mammal transmission. Whether such fitness enhancing variants get selected in nature is entirely dependent on random events, frequency of infection in the new species and time. Given the continued infection of humans with avian H7N9 viruses, the risk of adaptation and pandemic emergence remains plausible. Therefore, surveillance and risk assessment of H7N9 viruses in poultry along with monitoring infections in humans should remain a focus of pandemic preparedness efforts.

## Materials and methods

### Cells

MDCK epithelial cells, African green monkey kidney epithelial (Vero) cells and human embryonic kidney (HEK 293T) epithelial cells were obtained from the American Type Culture Collection. MDCK cells were maintained in MEM containing 5% heat-inactivated fetal bovine serum (FBS) and 0.5% penicillin–streptomycin. Vero cells were maintained in DMEM (Gibco) containing 10% heat-inactivated FBS and 2 mM L-glutamine. HEK 293T cells were maintained in Opti-MEM (Gibco, NY) supplemented with 10% heat-inactivated FBS. All the cells were maintained at 37 °C in 5% CO_2_.

### Biosafety, biosecurity and animal care

All the experiments were performed in a biosafety level 3 enhanced (ABSL3+) containment laboratory in accordance with USDA 9 CFR 121; 7 CFR 331. All the experiments were approved by the Institutional Biosafety Committee and the Dual Use Research of Concern Subcommittee at St Jude Children's Research Hospital. The work in this study was conducted prior to the current U.S. Government gain of function stop work order. The ABSL3+ facility has security and containment features that eliminate risks to humans and the environment^28^. The experiments were carried out by a trained personnel approved for work in ABSL3+ facilities. Animal studies were approved by the Animal Care and Use Committee of St Jude Children's Research Hospital. Food and water were provided *ad libitum* to all the animals.

### Viruses

The human H7N9 viruses A/Anhui/1/2013, A/Shanghai/1/2013, and A/Shanghai/2/2013 were obtained through the World Health Organization network. The virus stock used in the chicken and ferret experiments was grown in 10-day-old embryonated chicken eggs at 35 °C for 48 h.

### Virus infectivity

Virus stock was titrated by plaque assay in MDCK cells at 37 °C for 72 h with a 0.4% immunodiffusion grade agarose (MP Biomedical); and by EID_50_ assay in 10-day-old embryonated chicken eggs incubated at 35 °C for 48 h. Virus from chicken cloacal and oropharyngeal swabs was collected in 1-ml sterile phosphate-buffered saline (PBS) with antibiotics, titrated in 10-day-old embryonated chicken eggs at 35 °C for 48 h and titrated as EID_50_. Virus from ferret nasal washes and cell culture supernatants from replication kinetic experiments were titrated in MDCK cells at 37 °C for 72 h as TCID_50_. Virus titres (log_10_EID_50_ per ml and log_10_TCID_50_ per ml) in nasal washes, swabs, and tissues were estimated by the Reed and Muench method^52^ by using chicken red blood cells (0.5% in PBS). The lower limit of quantitation of the assays is 0.75 log_10_ EID_50_ per ml and 1.5 log_10_ TCID_50_ per ml.

### Experimental infection of chickens

Three 6-week-old, specific-pathogen-free chickens (*Gallus gallus domesticus*) were inoculated with 10^6 ^50% EID_50_ of A/Anhui/1/2013 (H7N9) influenza virus (0.5 ml) via natural routes (nares, trachea, and eyes). Cloacal and oropharyngeal samples were collected at 3, 5, 7 and 10 dpi with sterile cotton swabs, which were subsequently placed into 1 ml of PBS with antibiotics. Samples were stored at −80 °C for subsequent titration in 10-day-old embryonated chicken eggs as described above. The intravenous virus pathogenicity index was determined as described by Hulse *et al.*^53^ Infective allantoic fluid was diluted 1:10 in sterile PBS and 0.1 ml was injected intravenously into each of 10 6-week-old, specific-pathogen-free chickens. Chickens were monitored daily for 10 days for signs of illness or mortality.

### Experimental infection of ferrets

Young adult (3–4- month-old) male ferrets (*Mustela putorius furo*; Triple F Farms) that were serologically negative by hemagglutination inhibition assay for currently circulating A(H1N1), A(H3N2) and B human influenza viruses were used in the experiments. Six ferrets per group were inoculated intranasally with 10^6^ TCID_50_ of A/Anhui/1/2013 influenza virus in 0.5 ml PBS under isoflurane anaesthesia. On 3 and 5 dpi, three ferrets per group were humanely killed. Virus in the nasal cavities, tracheas, lungs, brains and the small and large intestines was titrated in MDCK cells as the TCID_50_. Excised tissue samples preserved in 10% neutral buffered formalin (Thermo Scientific) were paraffin-embedded, sliced into 5-μm-thick sections and stained with hematoxylin and eosin or with immunohistochemical stains for influenza A virus nucleoprotein, pneumocyte type II cells (surfactant protein c), macrophages (ionized calcium-binding adapter molecule 1) and neutrophils (myeloperoxidase). To collect nasal washes, ferrets were anaesthetised by intramuscular administration of ketamine HCl (25 mg kg^−1^) and sterile PBS with antibiotics (0.5 ml) was instilled into each nostril. The resultant washes were collected into a sterile cup. Samples were titrated by using the TCID_50_ assay in MDCK cells as described above.

### Virus transmission in ferrets

Two rounds of transmission experiments were performed. During round 1, four ferrets were inoculated intranasally with 10^6^ TCID_50_ of virus in 0.5 ml PBS under isoflurane anaesthesia and placed in separate cages with directed air flow. At 1 dpi, four naïve ferrets were placed in each of the inoculated ferrets' cages (DC) and another four ferrets were placed in AC in an adjacent cage separated from the inoculated ferrets' cage by a perforated divider with controlled air flow.

For the second round of experiments (secondary respiratory droplet transmission), six naïve ferrets were inoculated with a nasal wash sample containing ‘AC' virus obtained at 10 dpi (day 9 post contact) from the ferret infected via AC in the previous round of experiments. The nasal wash was diluted in PBS to a titre of ∼10^6^ TCID_50_. At 1 dpi, three naïve ferrets were placed in AC in an adjacent cage as described above. Body weight and clinical signs were monitored for 21 dpi. Virus was titrated in nasal washes collected from inoculated and AC ferrets on 1 dpi and then every other day for 14 dpi or until no virus shedding can be detected for two consecutive sneezes as described above.

### Serologic testing

Seroconversion was tested at 20 days post contact or at 21 dpi. Serum was treated with receptor-destroying enzyme (Seiken) overnight at 37 °C to destroy non-specific inhibitors, heat-inactivated at 56 °C for 30 min, and tested by hemagglutination inhibition assay with A/Anhui/1/2013 (H7N9) virus and 0.5% chicken red blood cells as described previously^54^.

### Deep amplicon sequencing and genetic analysis

Viral RNA was extracted by using an RNeasy kit (Qiagen); reverse-transcriptase PCR was then performed by using a SuperScript III first-strand synthesis kit (Invitrogen) with the Uni12 influenza primer. Multiplex PCR of all the eight gene segments was conducted by using PCR Supermix HiFi (Invitrogen) with the Uni12/13 and influenza A polymerase segment primers under previously described thermal cycler conditions^55^. PCR products were purified on a spin column (Qiagen) and quantified by using the Quant-It Pico Green kit (Invitrogen). DNA libraries were prepared by using NEXTera XT DNA-Seq library prep kits (Illumina) with 96 dual-index barcodes according to the manufacturer's instructions. Pooled libraries were sequenced on an Illumina MiSeq Personal Genome Sequencer, using 150 base-pair paired-end reads.

Genomic sequences were analysed by using a custom work-flow protocol. In brief, sequence reads were filtered for quality scores by using the Nextera adapter trimming protocol, discarding low-quality terminal bases with PHRED scores >20. A sample of 100,000 high-quality read pairs were aligned to the full set of complete NCBI influenza genomes in an exhaustive, competitive fashion to identify potential contaminating samples. Two rounds of alignment were performed: one pairwise alignment and one single-end alignment of unmapped reads from the first round. Genomes with at least one segment with a coverage >0.7 were selected and used to populate a new reference set. The full set of high-quality read pairs was aligned to H7N9 and this reduced reference set, by using the same approach. Genomic variations in H7N9 were detected by using an in-house single nucleotide variants (SNV) detection pipeline that considers base quality of a putative SNV, neighbourhood base quality, strand bias of SNV and several other factors. Heat maps were generated by using the R programming software package (R Core Team, 2013). Mean variant frequency for each sample was calculated by using positions that are variable in at least one of the examined samples/reads. If a site had a minor variant frequency >0 in any of the samples, it was included in the numerator; the denominator of the proportion was the total number of variable sites that was variable in all samples.

### Polymerase activity assays

Viral ribonucleoprotein complex genes, including NP, PA, PB1 and PB2 of A/Anhui/1/2013 (H7N9) virus, were cloned into the pHW2000 plasmid to test the viral polymerase activity^56^. The PB1-D76N, PB2-K627E and NP-I365V mutations (primers listed in Supplementary Table 6) were generated in pHW2000 plasmids by using a site-directed mutagenesis system (Agilent Technologies). The polymerase activity of the mutant and wild-type polymerase complexes was measured as previously described^57^. In brief, HEK 293T cells were transfected with 0.1 μg each of pHW2000-PB2, pHW2000-PB1, pHW2000-PA, pHW2000-NP, pHW72-Luci and pCMV-β-gal plasmids in 24-well-plates by using TransIT-LT1 transfection reagent (Mirus Bio). After 24 h, the cells were washed with PBS and lysed for 30 min with 100 μl of lysis buffer (Promega). The cells were then collected and the luciferase activity was assayed in triplicate by using the Promega luciferase assay system. Results were normalized to β-galactosidase activity.

### pH of HA activation

The pH of HA protein activation was measured by syncytium assay, as previously described^58^. In brief, Vero cells were inoculated with H7N9 influenza viruses (A/Anhui/1/2013, A/Shanghai/1/2013 and A/Shanghai/2/2013) at a multiplicity of infection of 5 TCID_50_ per cell and incubated at 37 °C for 18 h to allow cell surface expression of the HA protein. Cells were then washed with PBS and treated with 5 mg ml^−1^ of TPCK (L-tosylamido 2-phenylethyl chloromethyl ketone)-treated trypsin (Worthington) for 5 min, followed by neutralization with FBS. Cells were then washed twice with PBS and pulsed with PBS buffers adjusted to 0.1 pH increments by using 0.1 M citric acid. The highest pH at which syncytia were observed was recorded as the pH of HA activation.

### NA activity

The activity of cell surface viral NA proteins was assayed as previously described^59^. In brief, the mutations E-to-K at residue 73 and I-to-V at residue 300 were introduced individually or in combination into the NA gene carried in a pHW2000 plasmid by using a site-direct mutagenesis kit (Agilent Technologies), followed by the deletion of the *Cla*I site and subcloning of the product into a pCAGGS expression plasmid, using primers with overhangs that include *Cla*I and *Nhe*I restriction sites (Supplementary Table 6). An HA epitope tag was introduced into the carboxy-terminus of the NA gene during the subcloning step using primers listed in Supplementary Table 6.

To assess the NA activity, Vero cells were transfected with the HA-tagged NA plasmids carrying the mutations of interest (NAI300V and NAE73K), WT plasmid and an empty pCAGGS expression plasmid (mock) by using TransIT-LTI (Mirus Bio) transfection reagents. After incubation of the transfected cells for 20 h at 37 °C, cells were collected and resuspended in assay buffer (15 mM MOPS, 145 mM sodium chloride, 2.7 mM potassium chloride, and 4.0 mM calcium chloride, adjusted to pH 7.4 and supplemented with 2% heat-inactivated FBS). Fifty microlitres of the suspension was incubated with an equal volume of 2′-(4-methylumbelliferyl)-α-D-*N*-acetylneuraminic acid (MUNANA; Sigma-Aldrich) substrate (final concentration, 100 μM) for 30 min at 37 °C. The reaction was quenched with 100 μl stop solution (150 mM sodium hydroxide in 83% ethanol), and the fluorescence intensity of the released 4-methyl-umbelliferone was measured on a Synergy 2 multi-mode microplate reader (BioTek Instruments) with excitation and emission wavelengths of 360 and 460 nm, respectively. NA activity was quantified as fluorescence above the background fluorescence of mock-transfected cells, expressed in picomoles of 4-methyl-umbelliferone product.

To confirm the cell surface expression of NA protein, the Vero cells transfected as described above were collected, resuspended in 1X bovine serum albumin blocking buffer in Tris-buffered saline (Thermo Scientific) and stained with monoclonal anti-HA-FITC antibody (1:1,000 dilution; Sigma) for 1 h at 4 °C. HA-tagged NAs was then analysed by flow cytometry on a MACS Quant analyzer (Miltenyi Biotec). The mean fluorescence intensity above that of mock-transfected cells was quantified by FlowJo software.

### Receptor-binding affinity

Glycan array analysis was performed as previously described^60^, using glass slides containing 6 replicates of each of the 58 forms (Supplementary Table 7) of sialic acid-containing glycans, including terminal sequences, *N*-linked and *O*-linked glycans found on mammalian and avian glycoproteins and glycolipids^9^. Viruses were grown in MDCK cells, inactivated with β-propiolactone and concentrated on a sucrose gradient cushion. Viruses were applied to the microarray slides at a concentration of 128–256 HA U ml^−1^. Slides were incubated at room temperature for 1 h, washed with PBS and overlaid with rabbit or goat anti-H7 antibody (1:200) for 1 h, followed by the addition of 10 μg ml^−1^ anti-rabbit IgG (Alexa Fluor 488) or anti-goat IgG (Alexa Fluor 647; Invitrogen). Slides were washed and scanned on a ProScanArray Express HT (PerkinElmer) confocal slide scanner to quantify the bound virus.

### Virus rescue and replication kinetics in MDCK cells

The plasmids encoding the eight genes of A/Anhui/1/2013 (H7N9) virus cloned into plasmid vector pHW2000 have been described in ref. 61. Amino-acid changes were introduced by using the QuikChange site-directed mutagenesis kit (Agilent Technologies), as described above. Recombinant (rg) rg-A/Anhui/1/2013 viruses (WT), rg-A/Anhui/1/2013 (NA_E73K_), rg-A/Anhui/1/2013 (NA_I300V_), rg-A/Anhui/1/2013 (NA_E73K_+NA_I300V_), rg-A/Anhui/1/2013 (PB1_D76N_), rg-A/Anhui/1/2013 (NP_I365V_), rg-A/Anhui/1/2013 (PB1_D76N_+NP_I365V_) and rg-A/Anhui/1/2013 (NA_E73K_+NA_I300V_ +PB1_D76N_+NP_I365V_) were generated by co-transfecting 293T cells (TransIT-LT1, Mirus Bio) with the appropriate eight plasmids and the virus stocks were prepared in MDCK cells. The presence of the amino-acid substitutions of interest in the recombinant viruses were verified by Sanger sequencing.

To determine single-step growth curves, confluent monolayers of MDCK cells were infected with reverse-genetic viruses at an identical multiplicity of infection of 1 TCID_50_ per cell at 37 °C. After 1 h of incubation, the cells were washed with 0.9% aqueous NaCl solution (pH 2.2) to remove unbound virus particles, washed for three times with PBS to adjust the pH and overlaid with infection medium (MEM with 0.3% BSA; 1 μg ml^−1^ TPCK-treated trypsin). Supernatants were collected at 2, 4, 6, 8, 10, 12 and 24 h post inoculation and stored at −80 °C for titration in MDCK cells as described above. To quantify the total amount of virus produced over the course of the experiment, we computed the area under virus titre hour curve from 0 to 24 h (AUC_0–24_), using peak virus titres at 0, 2, 4, 6, 8, 10, 12 and 24 h post inoculation (log_10_TCID_50_ per ml) by GraphPad software.

### Statistical analysis

GraphPad Prism 5 software (GraphPad Software Inc.) was used for statistical analysis. Two-way analysis of variance was used to compare the groups. Two-tailed Mann–Whitney *U-*test was used to compare the genetic diversity. *P* values <0.05 were considered to indicate a statistically significant difference.

## Author contributions

H.Z. and T.B. designed and performed experiments, and drafted the manuscript; R.C., M.E., G.W. and J.Z. performed computational analysis; B.S.K., J.C.P., R.McB., M.-S.S., J.C.C., J.S., E.K., M.H. and T.F. performed the experiments. J.E.R. analysed the pathology data. J.B., Hu.Z., Y.G., S.S.-C., S.K., R.G.W., E.A.G. and C.J.R. designed the influenza virus infection experiments and contributed to writing the manuscript. R.J.W. designed and supervised the study, critically reviewed and revised the manuscript.

## Additional information

**How to cite this article:** Zaraket, H. *et al.* Mammalian adaptation of influenza A(H7N9) virus is limited by a narrow genetic bottleneck. *Nat. Commun.* 6:6553 doi: 10.1038/ncomms7553 (2015).

## Supplementary Material

Supplementary Information Supplementary Figures 1-5 and Supplementary Tables 1-7

Supplementary Dataset 1 Synonymous and non-synonymous mutations detected in chicken isolates

Supplementary Datatset 2 Synonymous and non-synonymous mutations detected in ferret isolates

## Figures and Tables

**Table 1:** Titres of A/Anhui/1/2013 (H7N9) influenza virus in chicken oropharyngeal swabs.

**Dpi**	**No. shedding/total**	**Mean virus titre (log**_**10**_**EID**_**50**_ **per ml**^−1^±s.d.)
3	4/4	3.75±1.33
5	4/4	3.94±1.54
7	2/4	1.50±0.00
10	0/4	<0.75*

Dpi, days post inoculation; No., number.

^*^<0.75, below the lower limit of detection. All the cloacal samples were below the lower limit of detection.

**Table 2:** The effect of the identified substitutions in the NA and polymerase genes on the total amount of influenza virus produced over the course of the experiment using replication kinetic assays in MDCK cells.

**rg-A/Anhui/1/2013 (H7N9) influenza virus**	**AUC**_**0–24**_ **(mean log**_**10**_**TCID**_**50**_ **per ml**^−1^±s.d.)	***P*** value*
WT	80.38±3.11	NA
NA_E73K_	63.88±2.29	<0.05
NA_I300V_	76.20±2.26	0.07
PB1_D76N_	81.10±3.79	0.78
NP_I365V_	77.19±2.20	0.15
NA_E73K_+ NA_I300V_	71.13±2.07	<0.05
PB1_D76N+_ NP_I365V_	72.25±1.67	<0.05
NA_E73K_+ NA_I300V_+PB1_D76N+_ NP_I365V_	69.19±3.74	<0.05

NA, not applicable.

AUC_0–24_,the area under virus titre hour curve from 0 to 24 h post inoculation. Values are mean and s.d. from two independent experiments (*n*=4).

^*^P values were compared with WT virus by two-way analysis of variance.
