# Supplementary material for: Mammalian adaptation of influenza A(H7N9) virus is limited by a narrow genetic bottleneck
Source: Nat Commun. 2015 Apr 8;6:6553. doi: 10.1038/ncomms7553 (PMC4403340; doi:10.1038/ncomms7553)
Supplement: Supplementary Information — Supplementary Figures 1-5 and Supplementary Tables 1-7 [file ncomms7553-s1.pdf]

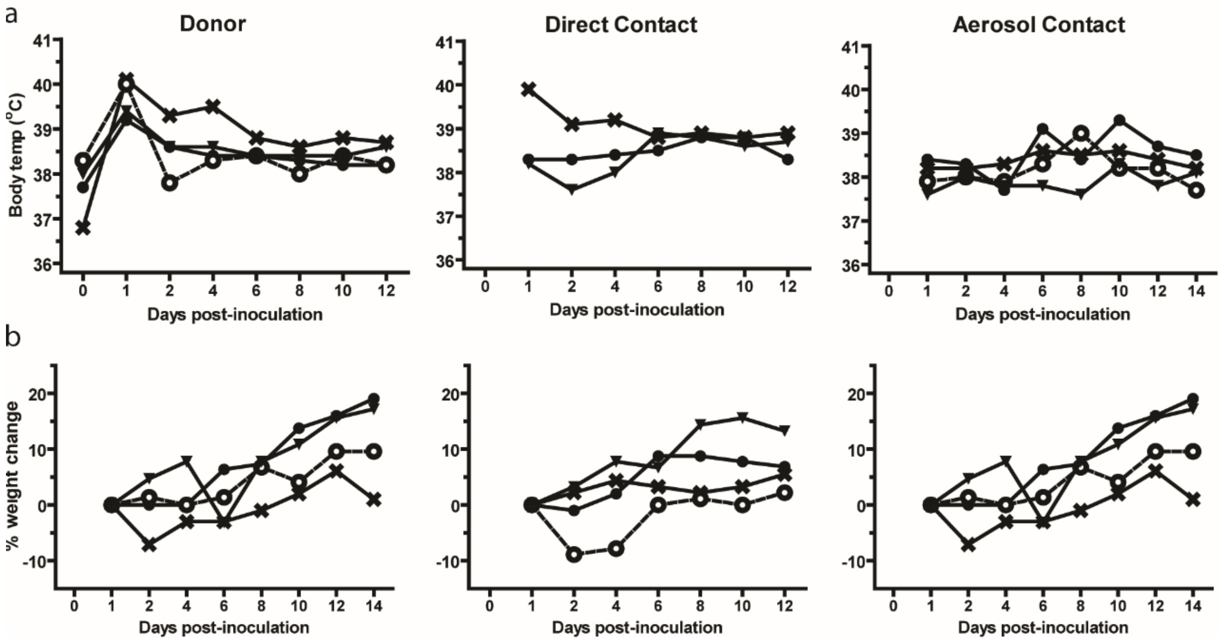

Supplementary Figure 1 | Weight and body temperature of ferrets inoculated with A/Anhui/1/2013 (H7N9) influenza virus. (**a**) Body temperature and (**b**) weight change of ferrets after intranasal inoculation with 10^6 TCID50 of A/Anhui/1/2013 (H7N9) influenza virus, direct contact, and aerosol contact (*n* = 4 per group). Body temperature is shown for only 3 direct contact ferrets, as one temperature probe was faulty.

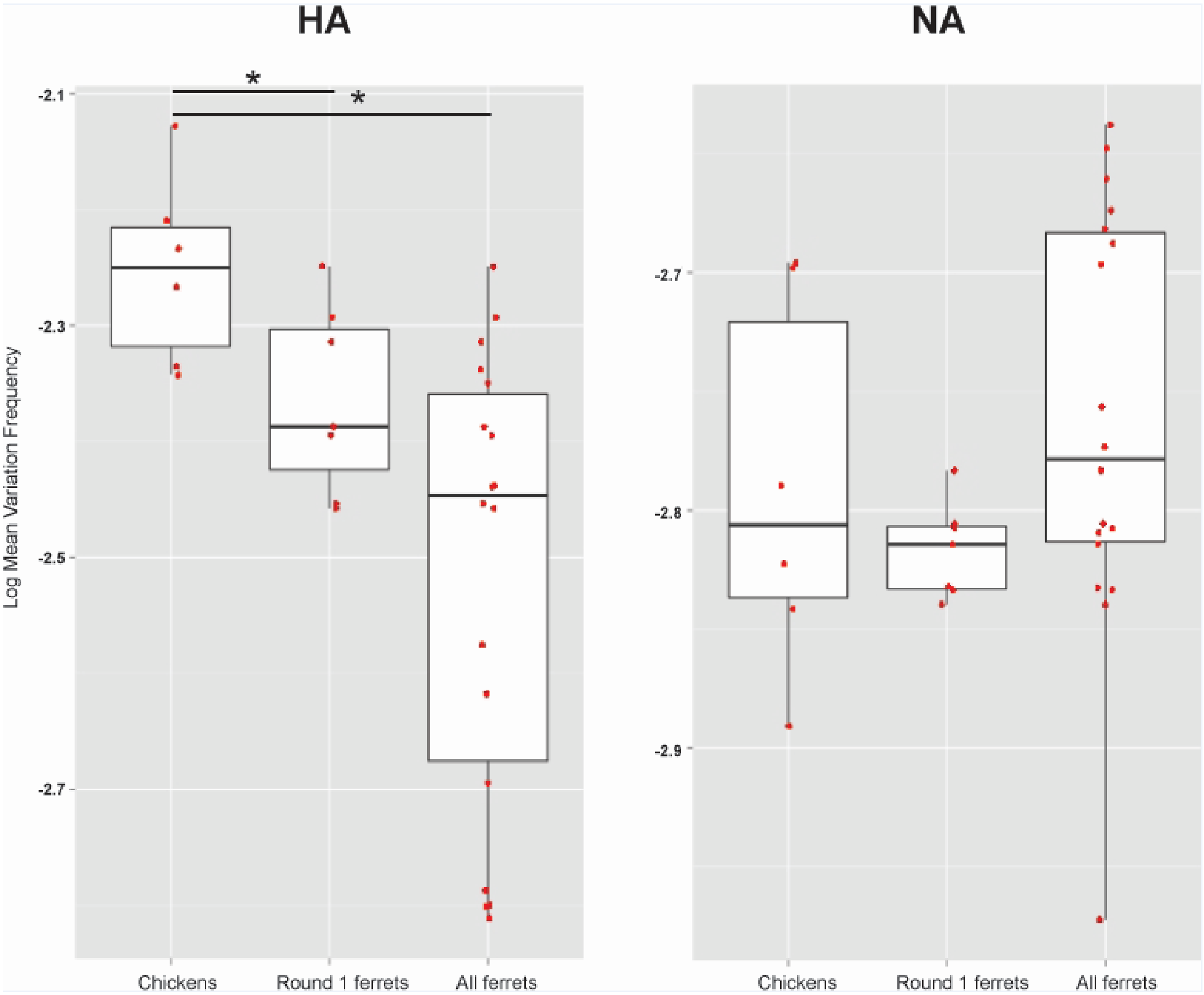

Supplementary Figure 2 | Mean variation frequencies of HA and NA genes after virus replication in chickens and ferrets. "Round 1 ferrets" were inoculated with parental A/Anhui/1/13 (H7N9) influenza virus; "all ferrets" include donor and contact ferrets in rounds 1 and 2. Statistical comparisons were performed by using two-tailed Mann-Whitney U test, **P* < 0.05.

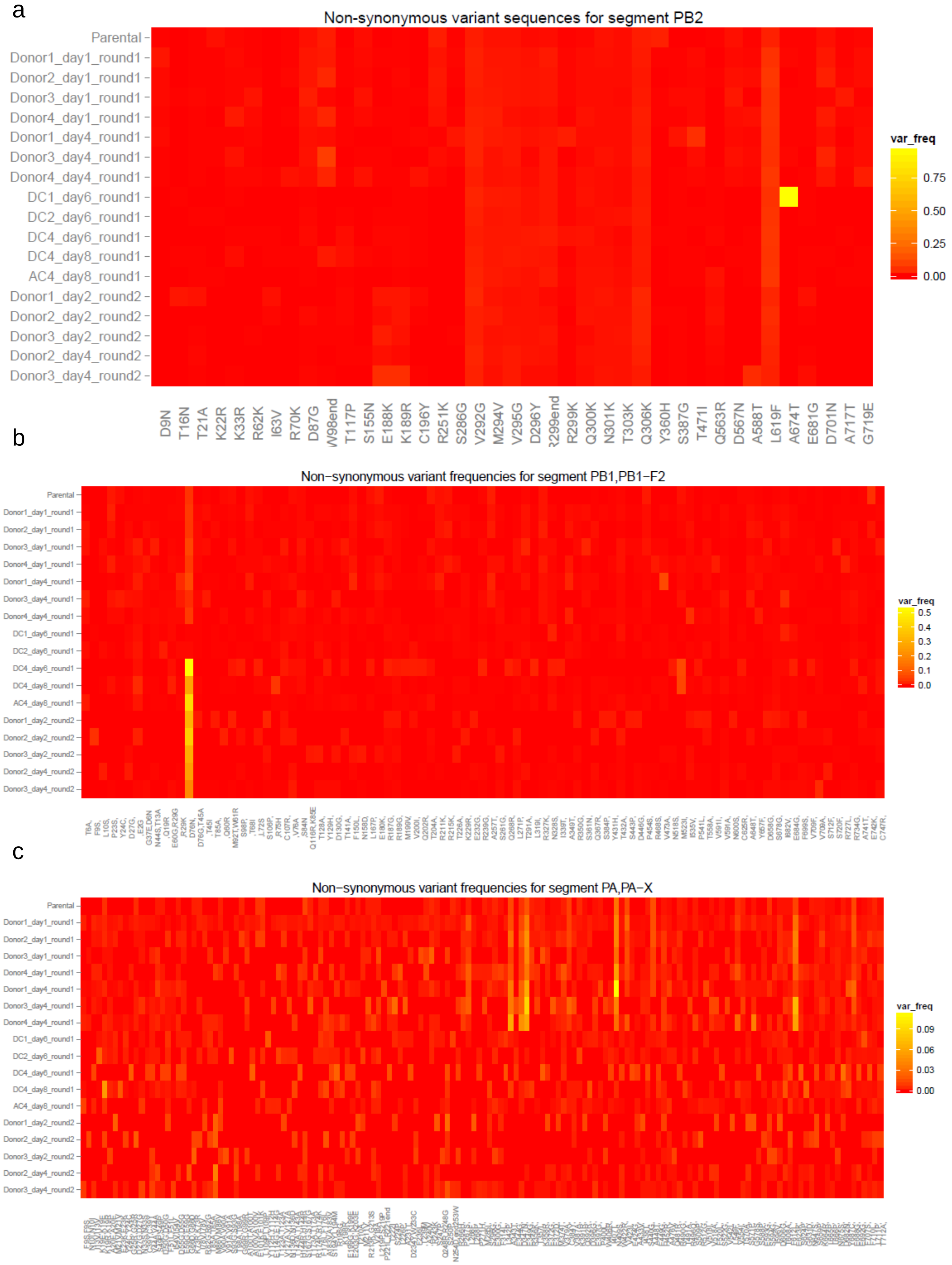
a
Non-synonymous variant sequences for segment PB2
Parental
Donor1_day1_round1
Donor2_day1_round1
Donor3_day1_round1
Donor4_day1_round1
Donor1_day4_round1
Donor3_day4_round1
Donor4_day4_round1
DC1_day6_round1
DC2_day6_round1
DC4_day6_round1
DC4_day8_round1
AC4_day8_round1
Donor1_day2_round2
Donor2_day2_round2
Donor3_day2_round2
Donor2_day4_round2
Donor3_day4_round2
D9N T16N T21A K22R K33R R62K I63V R70K D87G W98end T117P S155N E188K K189R C196Y R251K S286G V292G M294V V295G D296Y 299end R299K Q300K N301K T303K Q306K Y360H S387G T471I Q563R D567N A588T L619F A674T E681G D701N A717T G719E
var_freq
0.75
0.50
0.25
0.00
b
Non-synonymous variant frequencies for segment PB1,PB1-F2
var_freq
0.5
0.4
0.3
0.2
0.1
0.0
c
Non-synonymous variant frequencies for segment PA,PA-X
var_freq
0.09
0.06
0.03
0.00

d

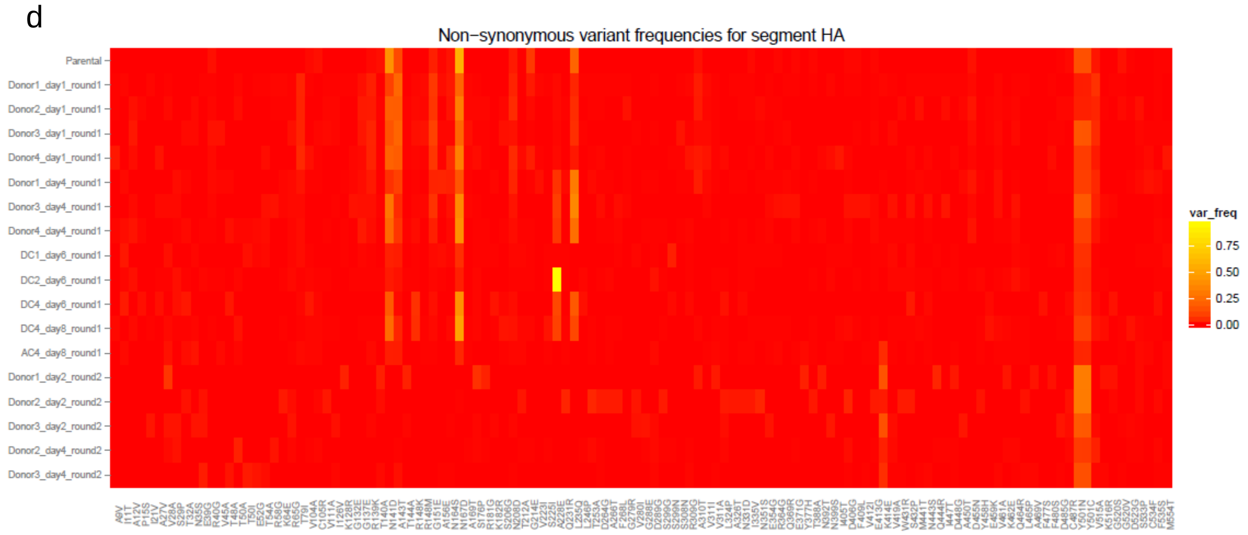
Non−synonymous variant frequencies for segment HA
Parental
Donor1_day1_round1
Donor2_day1_round1
Donor3_day1_round1
Donor4_day1_round1
Donor1_day4_round1
Donor3_day4_round1
Donor4_day4_round1
DC1_day6_round1
DC2_day6_round1
DC4_day6_round1
DC4_day8_round1
AC4_day8_round1
Donor1_day2_round2
Donor2_day2_round2
Donor3_day2_round2
Donor2_day4_round2
Donor3_day4_round2
var_freq
0.75
0.50
0.25
0.00

e

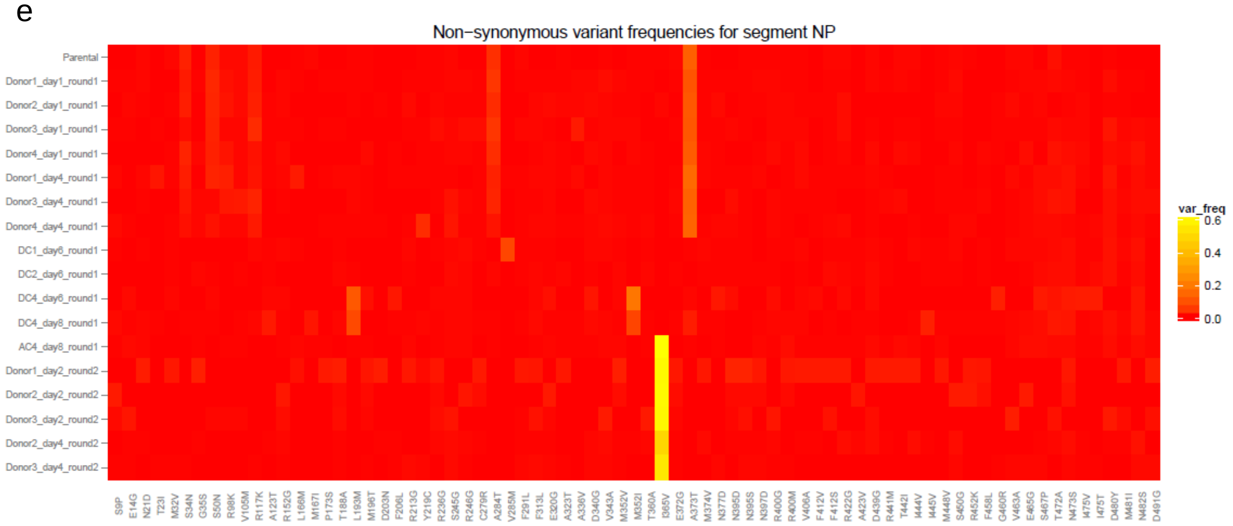
Non−synonymous variant frequencies for segment NP
Parental
Donor1_day1_round1
Donor2_day1_round1
Donor3_day1_round1
Donor4_day1_round1
Donor1_day4_round1
Donor3_day4_round1
Donor4_day4_round1
DC1_day6_round1
DC2_day6_round1
DC4_day6_round1
DC4_day8_round1
AC4_day8_round1
Donor1_day2_round2
Donor2_day2_round2
Donor3_day2_round2
Donor2_day4_round2
Donor3_day4_round2
var_freq
0.6
0.4
0.2
0.0
S9P E14G N21D T23I M32V S34N G35S S50N R98K V105M R117K A123T R152G L166M M167I P173S T188A L193M M196T D203N F206L R213G Y219C R236G S245G R246G C279R A284T V285M F291L F313L E320G A323T A336V D340G V343A M352V M352I T360A I365V E372G A373T M374V N377D N395D N395S N397D R400G R400M V406A F412V F412S R422G A423V D439G R441M T442I I444V I445V M448V S450G R452K F458L G460R V463A E465G S467P T472A N473S I475V I475T D480Y M481I N482S D491G

f

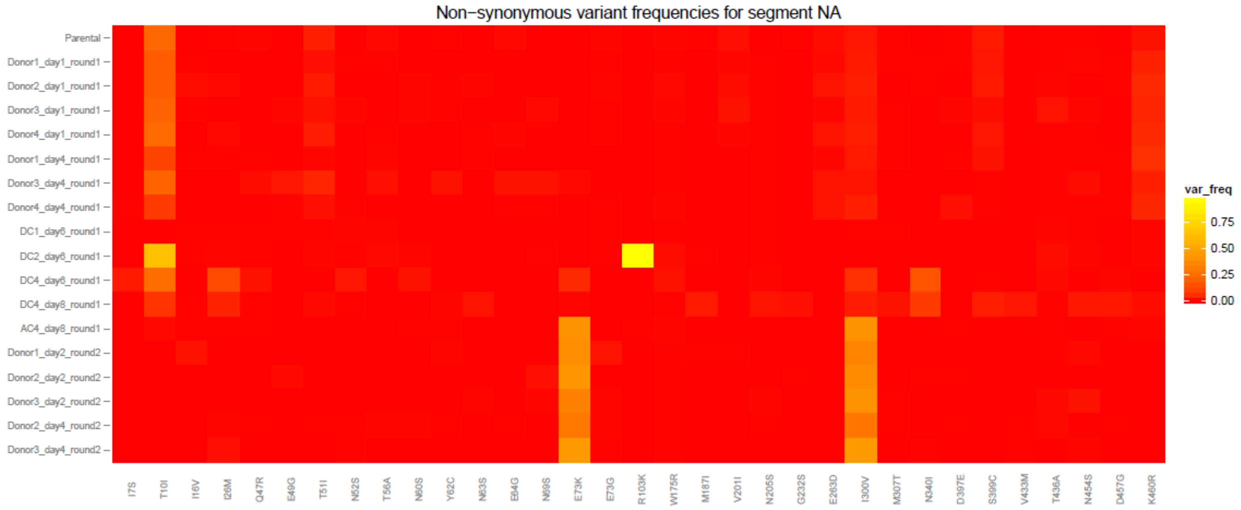
Non−synonymous variant frequencies for segment NA
Parental
Donor1_day1_round1
Donor2_day1_round1
Donor3_day1_round1
Donor4_day1_round1
Donor1_day4_round1
Donor3_day4_round1
Donor4_day4_round1
DC1_day6_round1
DC2_day6_round1
DC4_day6_round1
DC4_day8_round1
AC4_day8_round1
Donor1_day2_round2
Donor2_day2_round2
Donor3_day2_round2
Donor2_day4_round2
Donor3_day4_round2
var_freq
0.75
0.50
0.25
0.00
I7S T10I I16V I26M Q47R E49G T51I N52S T56A N60S Y62C N63S E64G N69S E73K E73G R103K W175R M187I V201I N205S G232S E263D I300V M307T N340I D397E S399C V433M T436A N454S D457G K460R

g

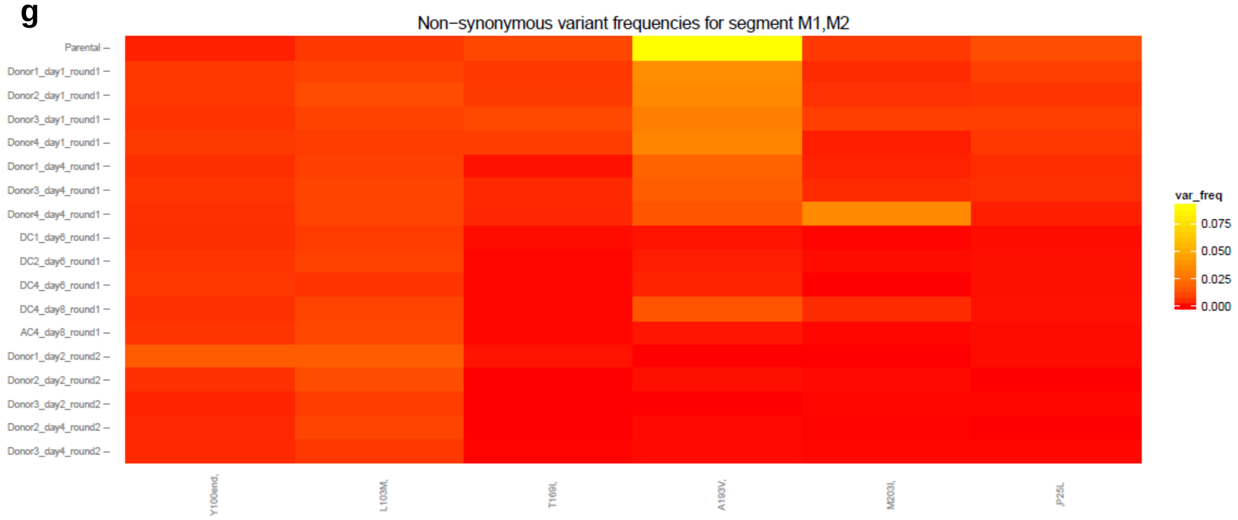

h

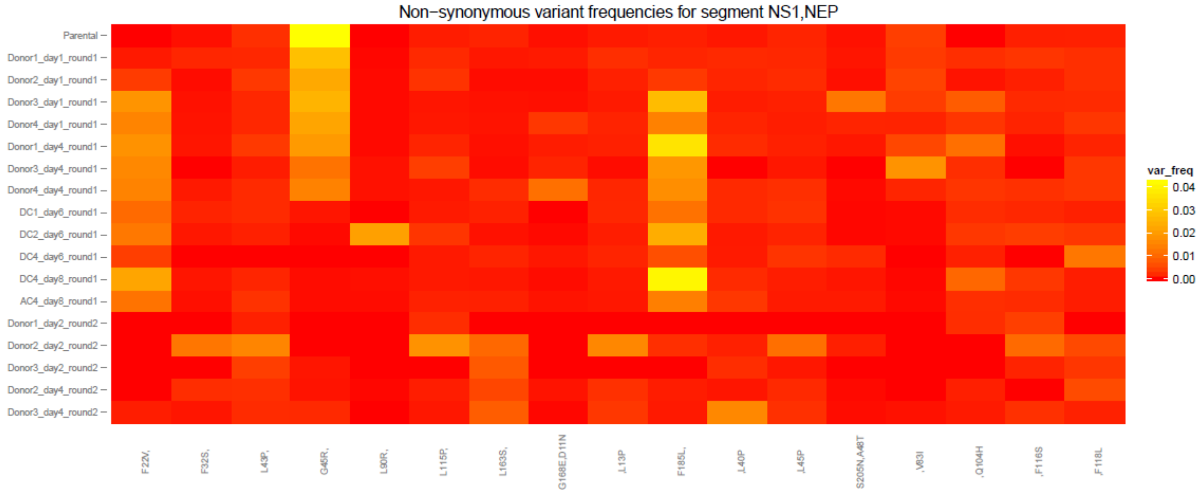

Supplementary Figure 3 | Heat map of variations in the whole genome of A/Anhui/1/2013 (H7N9) influenza virus in ferrets. Non-synonymous variant sequences for segments PB2 (a); PB1, PB1-F2 (b); PA, PA-X (c); HA (d); NP (e); NA (f); M1, M2 (g); and NS1, NEP (h) are shown. Parental virus is compared with viruses obtained in experimental rounds 1 and 2. Rows represent viruses from donor, direct contact (DC) and aerosol contact (AC) ferrets. Columns represent amino acid positions (counted from the start codon). Mutations in PA, M1, and NS1 genes are shown in black while those in PA-X, M2, and NS2 are in purple. Mutations with frequency > 0.01 are shown.

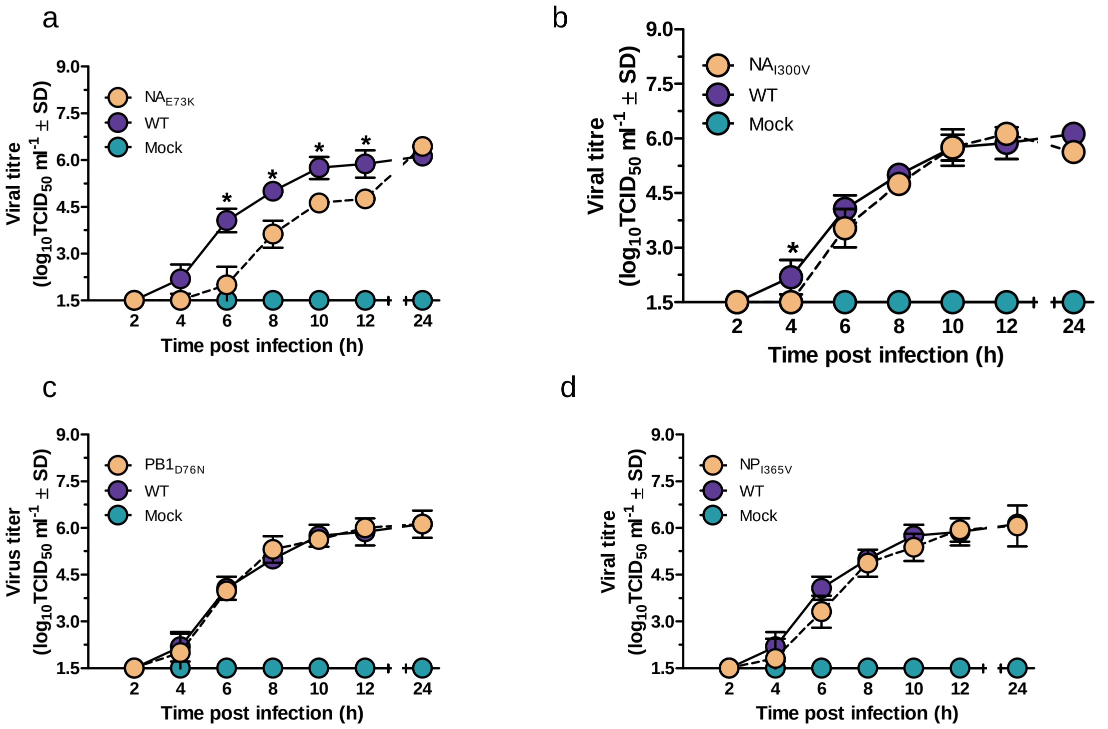

Supplementary Figure 4 | Effect of identified amino acid substitutions in the NA, PB1, and NP on replication kinetics of A/Anhui/1/2013 (H7N9) influenza viruses *in vitro*. Growth curves of the wild-type and the NA_{E73K} (**a**), NA_{I300V} (**b**), $PB1_{D76N}$ (**c**), and NP_{I365V} (**d**) rg-A/Anhui/1/2013 (H7N9) influenza viruses using MOI 1 $TCID_{50}$ per cell in MDCK cells at 37°C. Values are mean and SD from two independent experiments (n= 4). *<0.05 compared to WT virus by 2-way ANOVA.

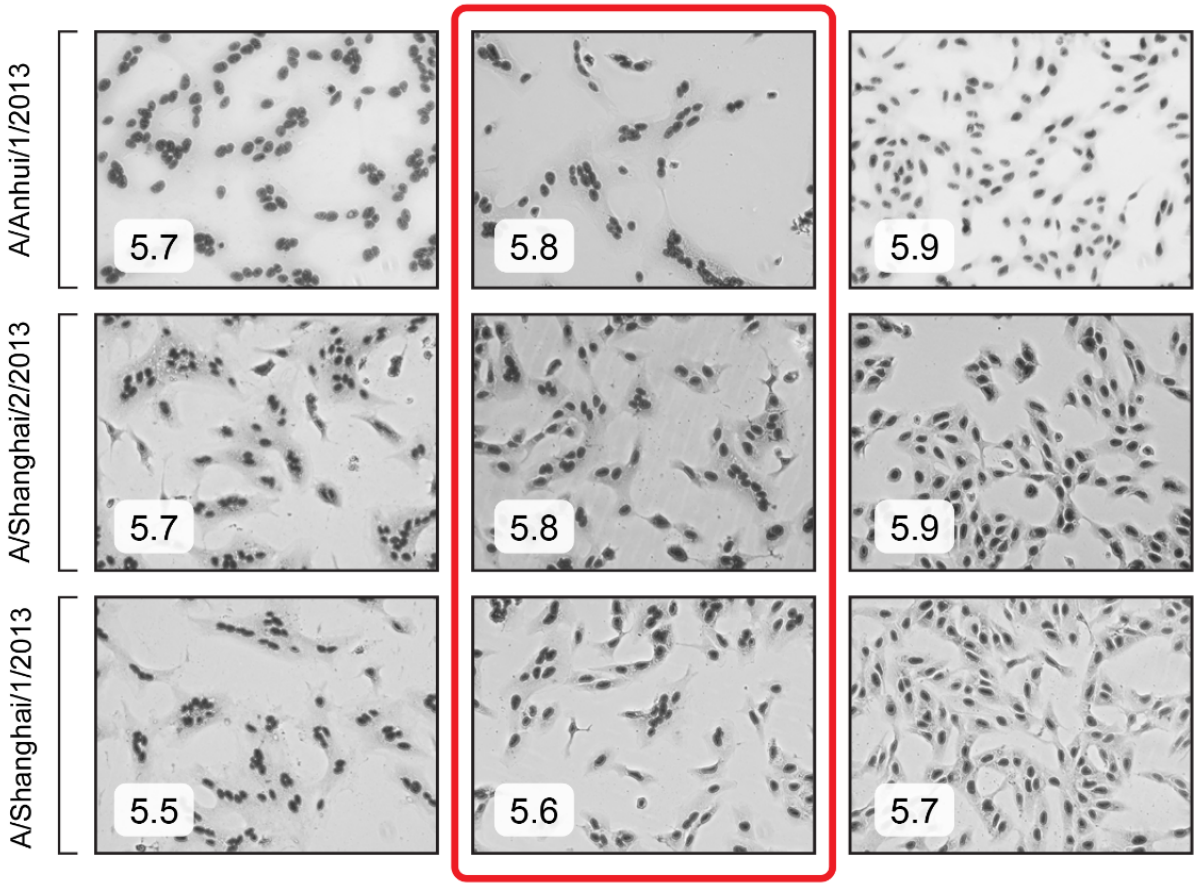

Supplementary Figure 5 | Activation pH of the HA proteins of H7N9 influenza viruses. Vero cells were inoculated with the indicated viruses at an MOI of 5 $TCID_{50/cell}$. Cells were then treated with pH buffers at the indicated pH values, and syncytium formation was monitored. The highest pH at which syncytia were observed (boxed in red) was recorded as the pH of HA activation. Data is representative of two independent experiments.

Supplementary Table 1 | Homology sequence analysis of A/Anhui/1/2013 (H7N9) influenza virus in comparison with H7N9 influenza viruses from birds and environment isolated during the first wave of the outbreak in China, 2013.

H7N9 virus (isolated form birds and environment)	Date of isolation	Segment[a]	Accession No.[b]	% Nucleotide sequence homology with A/Anhui/1/2013[c]	% Amino acid sequence homology with A/Anhui/1/2013
A/Chicken/Shanghai/S1053/2013	2013-04-03	PB2	EPI440682	99.8	99.8
		PB1	EPI440683	100.0	100.0
		PA	EPI440681	99.8	99.6
		HA	EPI440685	99.3	98.7
		NP	EPI440678	100.0	100.0
		NA	EPI440684	99.8	100.0
		MP	EPI440680	99.9	100.0
		NS	EPI440679	99.9	100.0
A/Chicken/Zhejiang/SD033/2013	2013-04-11	PB2	EPI457746	99.9	99.7
		PB1	EPI457747	100.0	100.0
		PA	EPI457745	100.0	100.0
		HA	EPI457749	99.9	100.0
		NP	EPI457742	100.0	100.0
		NA	EPI457748	99.9	99.8
		MP	EPI457744	98.3	99.3
		NS	EPI457743	99.9	100.
A/Environment/Shanghai/S1088/2013	2013-04-03	PB2	EPI440690	99.9	99.8
		PB1	EPI440691	100.0	100.0
		PA	EPI440689	99.4	98.7
		HA	EPI440693	99.4	99.1
		NP	EPI440686	100.0	100.
		NA	EPI440692	99.9	100.0
		MP	EPI440688	99.7	99.8
		NS	EPI440687	99.9	100.0
A/Pigeon/Shanghai/S1069/2013	2013-04-02	PB2	EPI440698	99.8	99.7
		PB1	EPI440699	100.0	100
		PA	EPI440697	99.8	99.6
		HA	EPI440701	99.8	100.0

		NP	EPI440694	98.8	99.7
		NA	EPI440700	99.8	99.8
		MP	EPI440696	99.8	100
		NS	EPI440695	96.9	99.4
A/Pigeon/Shanghai/S1423/2013	2013-04-03	PB2	EPI457626	99.9	99.8
		PB1	EPI457627	100.0	100.0
		PA	EPI457625	97.8	98.7
		HA	EPI457629	99.8	99.7
		NP	EPI457622	100.0	100.0
		NA	EPI457628	99.8	100.0
		MP	EPI457624	99.9	99.8
		NS	EPI457623	99.9	100.0

[a] PB2: RNA polymerase basic subunit 2; PB1: RNA polymerase basic subunit 1; PA: RNA polymerase acidic subunit; HA: haemagglutinin; NP: nucleoprotein; NA: neuraminidase; MP: matrix gene; NS: non-structural gene.

[b] Sequence data used in this table were downloaded from GISAID, Global Initiative on Sharing Avian Influenza Data (http://platform.gisaid.org)

[c] Homology analysis was performed using BioEdit software (http://www.mbio.ncsu.edu/bioedit/bioedit.html) Sequence Identity Matrix tool.

Supplementary Table 2 | Genetic diversity of A/Anhui/1/2013 (H7N9) influenza viruses in chickens.

**Animal number**	**Dpi**	**Gene segment**							
		**PB2**	**PB1**	**PA**	**HA**	**NP**	**NA**	**M**	**NS**
**(Parental virus)**	**N/A**	—[b]	—	—	N141D (0.4211), A143T (01250), N167D (0.5824), L235Q (0.2182), Y501N (0.1454), Y501C (0.1457)	A373T (0.1277)	T10I (0.2283)	—	—
1	3	N100D (0.4203), K627R (0.1687), L665P (0.125530625	N346S (0.125), V421A (0.3125), V609A (0.1355)	T210N (0.4737), A255G (0.3125), E377G (0.100)	N141D (0.6923), N167D (0.7945), Q444L (0.1477), Y501N (0.1874), Y501C (0.1884)	R26G (0.4809), R98K (0.1094), A284T (0.1975), A428T (0.1407)	T50K (0.1250), K139N (0.1677)	—[a]	—
	5	R70G (0.1102)	—	P28L (0.1070), S184N (0.7272), Q654stop (0.1698), R663G (0.1709)	N141D (0.4201), A143T (0.1203), G151E (0.1761), N167D (0.4350), Y501N (0.1935), Y501C (0.1935)	R99K (0.2261), A373T (0.1207), R446K (0.1058)	—	—	—

2	3	K48R (0.1020), W98stop (0.72), D275E (0.125)	—	E623K (0.3325)	N141D (0.4225), A143T (0.1509), N167D (0.5347), N208D (0.1339), A310T (0.1724), G475D (0.36951), Y501N (0.1598), Y501C (0.1598)	—	—	—	—
	5	—	—	—	N141D (0.6972), N167D (0.5634), Y501Y (0.1596), Y501C (0.1586)	—	T10I (0.2241)	M165I (0.1438), M192I (0.1846), A193V (0.1969)	—
3	3	M90T (0.2526)	K54E (0.6257), M523I (0.6470), Q569K (0.1161)	Y110H (0.1724)	N141D (0.7217), N167D (0.6614), L235Q (0.8971), Y501N (0.2537), Y501C (0.2529), G520V (0.1451)	A284T (0.1267),	—	—	—

5	—	F94L (0.1726), L108F (0.1274)	—	N141D (0.2786), N167D (0.3247), L235Q (0.1111), K414R (0.1111), Y501N (0.46939), Y501C (0.4694)	A373T (0.1265)	S119P (0.1097)	—	—

Three 6-week-old, specific pathogen–free chickens were inoculated with 10^6 EID_{50} of virus in 0.5 ml PBS via natural routes (nares, trachea, eyes). RNA was extracted from oropharyngeal swabs on the indicated days post-inoculation (dpi) and influenza virus genes were amplified and sequenced. Shown are all amino acid variations that differed from the consensus parental sequence and were present at a frequency ≥ 0.1.

—, no nonsynonymous mutations were observed at a frequency ≥ 0.1.

Supplementary Table 3 | Ferret serum hemagglutination inhibition titers.

A/Anhui/1/2013 (H7N9) virus	Serum HI titer (HAU 50 μl^{-1})											
	After inoculation				After direct contact				After aerosol contact			
	**D1**	**D2**	**D3**	**D4**	**DC1**	**DC2**	**DC3**	**DC4**	**AC1**	**AC2**	**AC3**	**AC4**
Parental	160	80	40	160	160	320	160	40	<	<	10	40
AC	80	160	<	ND	ND	ND	ND	ND	<	<	<	ND

Titers are reciprocal values. HAU, hemagglutinating units. D, donor; DC, direct contact; AC aerosol contact; <, below the lower limit of detection (10 HAU/50 μl); ND, not determined.

Supplementary Table 4 | Variation frequency of A/Anhui/1/2013 (H7N9) influenza virus after inoculation of ferrets and chickens.

Animal model	Experiment round	Group	Animal no.	DPI	Total variation frequency (log)	HA variation frequency (log)	NA variation frequency (log)
N/A	N/A	Parental virus	N/A	N/A	-2.905440162	-2.338603516	-2.972306897
Ferrets	1	Donor	D1	1	-2.806927772	-2.457879004	-2.833868038
				4	-2.742696441	-2.453907496	-2.805941954
			D2	1	-2.816327184	-2.387386966	-2.783279964
			D3	1	-2.769769832	-2.394822726	-2.81441596
				4	-2.776612542	-2.292597553	-2.807442559
			D4	1	-2.802506048	-2.24905896	-2.832470569
				4	-2.774978978	-2.313737707	-2.839594171
		Direct Contact	DC1	6	-2.772057833	-2.438035302	-2.756583119
			DC2	6	-2.748001965	-2.439238638	-2.773588496
			DC4	6	-2.841064242	-2.811259698	-2.637607146
				8	-2.779612706	-2.787387123	-2.696347657
		Aerosol Contact	AC4	8	-2.837547661	-2.349990787	-2.809618722
	2	Donor	D1-2	2	-2.832660393	-2.617216784	-2.660533406
			D2-2	2	-2.846847365	-2.575942667	-2.687619251
				4	-2.847458857	-2.694908903	-2.673835236
			D3-2	2	-2.851934700	-2.801427075	-2.681704602

				4	-2.841801531	-2.799644372	-2.647563697
Chickens	N/A	N/A	1	3	-2.634408252	-2.233465376	-2.841465687
				5	-2.675060293	-2.127884799	-2.891189157
			3	3	-2.67762697	-2.209500848	-2.697862833
				5	-2.736429542	-2.342424772	-2.822763484
			4	3	-2.659243758	-2.335177799	-2.695671659
				5	-2.764034125	-2.266838626	-2.789758423

Three- to 4-month-old male ferrets were inoculated intranasally with 10^6 $TCID_{50}$ of virus. Four 6-week-old, specific-pathogen-free chickens were inoculated with 10^6 50% egg infectious doses (EID_{50}). Viral genes were amplified and sequenced from RNA extracted from nasal washes (ferrets) or oropharyngeal swabs (chickens) on the indicated day pi. Mean variation frequency for each sample was calculated using positions that are variable in at least one of the examined samples. If a site has a minor variant frequency above zero in any of the samples this was included in the nominator and the denominator of the proportion was the total number of variable sites that was variable in all samples. Variation frequency was expressed as the negative log.

DPI, day post-inoculation when mutation was detected; N/A, not applicable.

Supplementary Table 5 | Genetic diversification of A/Anhui/1/2013 (H7N9) influenza viruses during two rounds of experiments in ferrets.

Experiment round	Ferret group	Ferret no.	DPI	Gene segment							
				PB2	PB1	PA	HA	NP	NA	MP	NS
1	(Parental virus)	N/A	N/A	–	–	–	N141D (0.4211), A143T (0.1250), N167D (0.5824), L235Q (0.2182), Y501N (0.1454), Y501C (0.1457)	A373T (0.1277)	T10I (0.2283)	–	–
	Donor	D1	1	–	–	–	A143T (0.2322), N167D (0.2251)	A373T (0.1036)	T10I (0.1838)	–	–
			4	–	–	N409S (0.1130)	N167D (0.1619), L235Q (0.3133)	A373T (0.1530)	T10I (0.1019)	–	–
		D2	1	–	–	–	N141D (0.1936), A143T (0.1922), N167D (0.3266)	A373T (0.1123)	T10I (0.1822)	–	–
		D3	1	–	–	–	N141D (0.1561), A143T (0.2241), N167D (0.2254), Y501N (0.1688), Y501C (0.1628)	A373T (0.1078)	T10I (0.2023)	–	–
			4	–	–	–	N141D (0.2861), A143T (0.1873), N167D (0.3769), L235Q (0.3691), Y501N (0.1782), Y501C (0.1782)	A373T (0.1371)	T10I (0.2098)	–	–
		D4	1	–	–	–	N141D (0.2087), N167D (0.3380)	A373T (0.1199)	T10I (0.2392)	–	–
			4	–	–	–	N141D (0.2391), N167D (0.4117), L235Q (0.2562)	A373T (0.1408)	–	–	–
	Direct Contact	DC1	6	A674T (0.9712)	–	–	–	–	–	–	–
	Direct Contact	DC2	6	–	–	–	A228E (0.9880)	–	T10I (0.6308),	–	–

									R103K (0.9576)		
		**DC4**	**6**	–	D76N (0.5316)	–	N141D (0.1818), N167D (0.4178), A228E (0.1173), L235Q (0.1737)	L193M (0.1111), M352I (0.1887)	T10I (0.2404), I26M (0.1266), N340I (0.1511)	–	–
			**8**	–	D76N (0.2793)	–	N141D (0.2464), N167D (0.5005), A228E (0.1004), L235Q (0.1174)	–	–	–	–
	**Aerosol Contact**	**AC4**	**8**	–	D76N (0.4320)	–	–	I365V (0.6387)	E73K (0.4058), I300V (0.4103)	–	–
**2**	**Donor**	**D1-2**	**2**	–	D76N (0.3177)	–	K414E (0.1563), Y501N (0.3182), Y501C (0.3182)	I365V (0.6163)	E73K (0.3941), I300V (0.3451)	–	–
	**Donor**	**D2-2**	**2**	–	D76N (0.3887)	–	Y501N (0.3137), Y501C (0.3137)	I365V (0.6236)	E73K (0.4348), I300V (0.3739)	–	–
			**4**	–	D76N (0.2604)	–	–	I365V (0.4976)	E73K (0.2933), I300V (0.2848)	–	–
		**D3-2**	**2**	–	D76N (0.2993)	–	K414E (0.1455), Y501N (0.1783), Y501C (0.1719)	I365V (0.6174)	E73K (0.3309), I300V (0.4053)	–	–
			**4**		D76N (0.2016)		Y501N (0.1449), Y501C (0.1439)	I365V (0.5547)	E73K (0.4471), I300V (0.4426)		

Three to 4-month-old male ferrets were inoculated intranasally with 10^6 $TCID_{50}$ of virus. Viral genes were amplified and sequenced from RNA extracted from nasal washes on the indicated day pi. Shown are all amino acid variations that differed from the consensus parental sequence and were present at a frequency of ≥ 0.1. Numbers in parentheses indicate the frequency at which each mutation occurred under each condition.

DPI, day post inoculation when mutation was detected; NA, not applicable; –, no nonsynonymous mutations were detected at a frequency ≥ 0.1.

Supplementary Table 6 | List of primers used in the study.

Primer	Description	Sequence (5' → 3')
N9 E73K F	E73K site-directed mutagenesis	CAAACATCACCAACATCCAAATGAAAGAGAGAACAAG
N9 E73K R	E73K site-directed mutagenesis	GAAATTCCTGCTTGTTCTCTCTTTCATTTGGATGTTG
N9 I300V F	I300V site-directed mutagenesis	GCAGGGCTCAAATAGACCAGTGGTTCAGATAGACCC
N9 I300V R	I300V site-directed mutagenesis	GTGTCATTGCTACTGGGTCTATCTGAACCACTGGTCTATTTG
N9 ClaI/- F	ClaI site deletion	GTGGACCAGCAATAGTATAGTTTCGATGTGTTCCAGTACAG
N9 ClaI/- R	ClaI site deletion	CCAGGAATTCTGTACTGGAACACATCGAAACTATACTATTGC
PB2 K627E F	K627E site-directed mutagenesis	CCATTTGCAGCAGCCCCGCCGGAGCAGAGTAGG
PB2 K627E R	K627E site-directed mutagenesis	CGGCGGGGCTGCTGCAAATGGTAATAGCTT
PB1 D76N F	D76N site-directed mutagenesis	ATTGATGGACCATTACCTGAGAACAACGAGCCGA
PB1 D76N R	D76N site-directed mutagenesis	CTCAGGTAATGGTCCATCAATTGGATTGAGT
I365V F	I365V site-directed mutagenesis	GCTATCCACTAGAGGGGTTCAAGTTGCTTCAAAT
I365V R	I365V site-directed mutagenesis	TTGAACCCCTCTAGTGGATAGCTGTCCTCTT
N9 ClaI F	N-terminal ClaI restriction site insertion for pCAAGS cloning	TTACCATCGATATGAATCCAAATCAGAAGATTC
N9 NheI HA-tag R	C-terminal NheI restriction site and HA-tag insertion	TTTCGCGCTAGCTTAAGCGTAATCTGGAACATCGTATGGGTAGAGGAAGTACT CTATTTTAGCC
N9 NheI R	C-terminal NheI restriction site insertion	TTTCGCGCTAGCTTAGAGGAAGTACTCTATTTTAGCC

Supplementary Table 7 | List of sialic acid-containing glycans used in glycan arrays

Chart number	Name	NeuAcα-	Type	Sulfated
1	Galβ(1-4)-GlcNAcβ-ethyl-NH_2	–	Type 2	
2	Galβ(1-4)-GlcNAcβ(1-2)-Manα(1-3)-[Galβ(1-4)-GlcNAcβ(1-2)-Manα(1-6)]-Manβ(1-4)-GlcNAcβ(1-4)-GlcNAcβ-Asn-NH_2	–	N	
3	NeuAca(2-3)-Galβ(1-4)-6-O-sulfo-GlcNAcβ-propyl-NH_2	3	Type 2	6S-GlcNAc
4	NeuAcα(2-3)-Galβ(1-4)-[Fucα(1-3)]-6-O-sulfo-GlcNAcβ-propyl-NH_2	3	Type 2	6S-GlcNAc
5	NeuAcα(2-3)-6-O-sulfo-Galβ(1-4)-GlcNAcβ-ethyl-NH_2	3	Type 2	6S-Gal
6	NeuAcα(2-3)-6-O-sulfo-Galβ(1-4)-[Fucα(1-3)]-GlcNAcβ-propyl-NH_2	3	Type 2	6S-Gal
7	NeuAcα(2-3)-Galβ(1-3)-6-O-sulfo-GlcNAcβ-propyl-NH_2	3	Type 1	6S-GlcNAc
8	NeuAca(2-3)-Galβ(1-4)-Glcβ-ethyl-NH_2	3	Lacto	
9	NeuAca(2-3)-Galβ(1-4)-GlcNAcβ-ethyl-NH_2	3	Type 2	
10	NeuAca(2-3)-Galβ(1-4)-GlcNAcβ(1-3)-Galβ(1-4)-GlcNAcβ-ethyl-NH_2	3	Type 2	
11	NeuAcα(2-3)-Galβ(1-4)-GlcNAcβ(1-3)-Galβ(1-4)-GlcNAcβ(1-3)-Galβ(1-4)-GlcNAcβ-ethyl-NH_2	3	Type 2	
12	NeuAca(2-3)-GalNAcβ(1-4)-GlcNAcβ-ethyl-NH_2	3	LacDiNAc	
13	NeuAca(2-3)-Galβ(1-3)-GlcNAcβ-ethyl-NH_2	3	Type 1	
14	NeuAca(2-3)-Galβ(1-3)-GlcNAcβ(1-3)-Galβ(1-4)-GlcNAcβ-ethyl-NH_2	3	Type 2	

15	NeuAca(2-3)-Galβ(1-3)-GlcNAcβ(1-3)-Galβ(1-3)-GlcNAcβ-ethyl-NH_2	3	Type 1
16	NeuAca(2-3)-Galβ(1-3)-GalNAcβ(1-3)-Gala(1-4)-Galβ(1-4)-Glcβ-ethyl-NH_2	3	Lacto
17	NeuAcα(2-3)-Galβ(1-3)-GalNAcα-Thr-NH_2	3	Core 1
18	NeuAcα(2-3)-Galβ(1-3)-[GlcNAcβ(1-6)]-GalNAcα-Thr-NH_2	3	Core 2
19	NeuAcα(2-3)-Galβ(1-4)-GlcNAcβ(1-6)-[Galβ(1-3)]-GalNAcα-Thr-NH_2	3	Core 2
20	NeuAcα(2-3)-Galβ(1-4)-GlcNAcβ(1-3)-Galβ(1-4)-GlcNAcβ(1-6)-[Galβ(1-3)]-GalNAcα-Thr-NH_2	3	Core 2
21	NeuAcα(2-3)-Galβ(1-4)-GlcNAcβ(1-3)-GalNAcα-Thr-NH_2	3	Core 3
22	NeuAcα(2-3)-Galβ(1-4)-GlcNAcβ(1-3)-Galβ(1-4)-GlcNAcβ(1-3)-GalNAcα-Thr-NH_2	3	Core 3
23	NeuAcα(2-3)-Galβ(1-4)-GlcNAcβ(1-3)-[NeuAcα(2-3)-Galβ(1-4)-GlcNAcβ(1-6)]-GalNAcα-Thr-NH_2	3	Core 4
24	NeuAcα(2-3)-Galβ(1-4)-GlcNAcβ(1-3)-Galβ(1-4)-GlcNAcβ(1-3)-[NeuAcα(2-3)-Galβ(1-4)-GlcNAcβ(1-3)-Galβ(1-4)-GlcNAcβ(1-6)]-GalNAcα-Thr-NH_2	3	Core 4
25	NeuAcα(2-3)-Galβ(1-4)-GlcNAcβ(1-2)-Manα(1-3)-[NeuAcα(2-3)-Galβ(1-4)-GlcNAcβ(1-2)-Manα(1-6)]-Manβ(1-4)-GlcNAcβ(1-4)-GlcNAcβ-Asn-NH_2	3	N
26	NeuAcα(2-3)-Galβ(1-4)-GlcNAcβ(1-3)-Galβ(1-4)-GlcNAcβ(1-2)-Manα(1-3)-[NeuAcα(2-3)-Galβ(1-4)-GlcNAcβ(1-3)-Galβ(1-4)-GlcNAcβ(1-2)-Manα(1-6)]-Manβ(1-4)-GlcNAcβ(1-4)-GlcNAcβ-Asn-NH_2	3	N
27	NeuAcα(2-3)-Galβ(1-4)-GlcNAcβ(1-3)-Galβ(1-4)-GlcNAcβ(1-3)-Galβ(1-4)-GlcNAcβ(1-2)-Manα(1-3)-[NeuAcα(2-3)-Galβ(1-4)-GlcNAcβ(1-3)-Galβ(1-4)-GlcNAcβ(1-3)-Galβ(1-4)-GlcNAcβ(1-2)-Manα(1-6)]-Manβ(1-4)-GlcNAcβ(1-4)-GlcNAcβ-Asn-NH_2	3	N

28	NeuAca(2-3)-[GalNAcβ(1-4)]-Galβ(1-4)-GlcNAcβ-ethyl-NH_2	3	Type 2	
29	NeuAca(2-3)-[GalNAcβ(1-4)]-Galβ(1-4)-Glcβ-ethyl-NH_2	3	Ganglio	
30	Galβ(1-3)-GalNAcβ(1-4)-[NeuAca(2-3)]-Galβ(1-4)-Glcβ-ethyl-NH_2	3	Ganglio	
31	NeuAca(2-3)-Galβ(1-4)-[Fuca(1-3)]-GlcNAcβ-propyl-NH_2	3	Type 2	
32	NeuAca(2-3)-Galβ(1-3)-[Fuca(1-4)]-GlcNAcβ(1-3)-Galβ(1-4)-[Fuca(1-3)]-GlcNAcβ-ethyl-NH_2	3	Type 2	
33	NeuAca(2-3)-Galβ(1-4)-[Fuca(1-3)]-GlcNAcβ(1-3)-Galβ(1-4)-[Fuca(1-3)]-GlcNAcβ-ethyl-NH_2	3	Type 2	
34	NeuAca(2-3)-Galβ(1-4)-[Fuca(1-3)]-GlcNAcβ(1-3)-Galβ(1-4)-[Fuca(1-3)]-GlcNAcβ(1-3)-Galβ(1-4)-[Fuca(1-3)]-GlcNAcβ-ethyl-NH_2	3	Type 2	
35	NeuGca(2-3)-Galβ(1-4)-GlcNAcβ-ethyl-NH_2	3-Gc	Type 2	
36	NeuAca(2-6)-Galβ(1-4)-6-O-sulfo-GlcNAcβ-propyl-NH_2	6	Type 2	6S-GlcNAc
37	NeuAca(2-6)-Galβ(1-4)-Glcβ-ethyl-NH_2	6	Lacto	
38	NeuAca(2-6)-Galβ(1-4)-GlcNAcβ-ethyl-NH_2	6	Type 2	
39	NeuAca(2-6)-Galβ(1-4)-GlcNAcβ(1-3)-Galβ(1-4)-GlcNAcβ-ethyl-NH_2	6	Type 2	
40	NeuAca(2-6)-Galβ(1-4)-GlcNAcβ(1-3)-Galβ(1-4)-GlcNAcβ(1-3)-Galβ(1-4)-GlcNAcβ-ethyl-NH_2	6	Type 2	
41	NeuAcα(2-6)-Galβ(1-4)-GlcNAcβ(1-3)-[NeuAcα(2-6)]-Galβ(1-4)-GlcNAcβ-ethyl-NH_2	6	Type 2	
42	NeuAca(2-6)-GalNAcβ(1-4)-GlcNAcβ-ethyl-NH_2	6	LacDiNAc	

43	NeuAcα(2-6)-[Galβ(1-3)]-GalNAcα-Thr-NH_2	6	Core 1
44	NeuAcα(2-6)-Galβ(1-4)-GlcNAcβ(1-6)-[Galβ(1-3)]-GalNAcα-Thr-NH_2	6	Core 2
45	NeuAcα(2-6)-Galβ(1-4)-GlcNAcβ(1-3)-Galβ(1-4)-GlcNAcβ(1-6)-[Galβ(1-3)]-GalNAcα-Thr-NH_2	6	Core 2
46	NeuAcα(2-6)-Galβ(1-4)-GlcNAcβ(1-3)-GalNAcα-Thr-NH_2	6	Core 3
47	NeuAcα(2-6)-Galβ(1-4)-GlcNAcβ(1-3)-Galβ(1-4)-GlcNAcβ(1-3)-GalNAcα-Thr-NH_2	6	Core 3
48	NeuAcα(2-6)-Galβ(1-4)-GlcNAcβ(1-3)-[NeuAcα(2-6)-Galβ(1-4)-GlcNAcβ(1-6)]-GalNAcα-Thr-NH_2	6	Core 4
49	NeuAcα(2-6)-Galβ(1-4)-GlcNAcβ(1-3)-Galβ(1-4)-GlcNAcβ(1-3)-[NeuAcα(2-6)-Galβ(1-4)-GlcNAcβ(1-3)-Galβ(1-4)-GlcNAcβ(1-6)]-GalNAcα-Thr-NH_2	6	Core 4
50	Galβ(1-4)-GlcNAcβ(1-2)-Manα(1-3)-[NeuAcα(2-6)-Galβ(1-4)-GlcNAcβ(1-2)-Manα(1-6)]-Manβ(1-4)-GlcNAcβ(1-4)-GlcNAcβ-Asn-NH_2	6	N
51	NeuAcα(2-6)-Galβ(1-4)-GlcNAcβ(1-2)-Manα(1-3)-[Galβ(1-4)-GlcNAcβ(1-2)-Manα(1-6)]-Manβ(1-4)-GlcNAcβ(1-4)-GlcNAcβ-Asn-NH_2	6	N
52	GlcNAcβ(1-2)-Manα(1-3)-[NeuAcα(2-6)-Galβ(1-4)-GlcNAcβ(1-2)-Manα(1-6)]-Manβ(1-4)-GlcNAcβ(1-4)-GlcNAcβ-Asn-NH_2	6	N
53	NeuAcα(2-6)-Galβ(1-4)-GlcNAcβ(1-2)-Manα(1-3)-[NeuAcα(2-6)-Galβ(1-4)-GlcNAcβ(1-2)-Manα(1-6)]-Manβ(1-4)-GlcNAcβ(1-4)-GlcNAcβ-Asn-NH_2	6	N
54	NeuAcα(2-6)-Galβ(1-4)-GlcNAcβ(1-3)-Galβ(1-4)-GlcNAcβ(1-2)-Manα(1-3)-[NeuAcα(2-6)-Galβ(1-4)-GlcNAcβ(1-3)-Galβ(1-4)-GlcNAcβ(1-2)-Manα(1-6)]-Manβ(1-4)-GlcNAcβ(1-4)-GlcNAcβ-Asn-NH_2	6	N

55	NeuAcα(2-6)-Galβ(1-4)-GlcNAcβ(1-3)-Galβ(1-4)-GlcNAcβ(1-3)-Galβ(1-4)-GlcNAcβ(1-2)-Manα(1-3)-[NeuAcα(2-6)-Galβ(1-4)-GlcNAcβ(1-3)-Galβ(1-4)-GlcNAcβ(1-3)-Galβ(1-4)-GlcNAcβ(1-2)-Manα(1-6)]-Manβ(1-4)-GlcNAcβ(1-4)-GlcNAcβ-Asn-NH_2	6	N
56	NeuGcα(2-6)-Galβ(1-4)-GlcNAcβ-ethyl-NH_2	6-Gc	Type 2
57	NeuAcα(2-3)-Galβ(1-4)-GlcNAcβ(1-2)-Manα(1-3)-[NeuAcα(2-6)-Galβ(1-4)-GlcNAcβ(1-2)-Manα(1-6)]-Manβ(1-4)-GlcNAcβ(1-4)-GlcNAcβ-Asn-NH_2	3/6	N
58	NeuAcα(2-6)-Galβ(1-4)-GlcNAcβ(1-2)-Manα(1-3)-[NeuAcα(2-3)-Galβ(1-4)-GlcNAcβ(1-2)-Manα(1-6)]-Manβ(1-4)-GlcNAcβ(1-4)-GlcNAcβ-Asn-NH_2	3/6	N
